# Persistent *Ascaris* Transmission Is Possible in Urban Areas Even Where Sanitation Coverage Is High

**DOI:** 10.1021/acs.est.2c04667

**Published:** 2022-10-26

**Authors:** Drew Capone, Troy Barker, Oliver Cumming, Abeoseh Flemister, Riley Geason, Elizabeth Kim, Jackie Knee, Yarrow Linden, Musa Manga, Mackenzie Meldrum, Rassul Nala, Simrill Smith, Joe Brown

**Affiliations:** † Department of Environmental and Occupational Health, School of Public Health, Indiana University, Bloomington, Indiana47401, United States; ‡ Department of Environmental Sciences and Engineering, Gillings School of Public Health, University of North Carolina at Chapel Hill, Chapel Hill, North Carolina27599, United States; § Department of Disease Control, London School of Hygiene and Tropical Medicine, LondonWC1E 7HT, U.K.; ∥ Department of Biology, University of North Carolina at Chapel Hill, Chapel Hill, North Carolina27599, United States; ⊥ Department of Civil and Environmental Engineering, Georgia Institute of Technology, Atlanta, Georgia30332, United States; # Ministério da Saúde, Instituto Nacional de Saúde Maputo, Maputo1102, Mozambique

**Keywords:** onsite, sanitation, Ascaris, pathogens, helminths

## Abstract

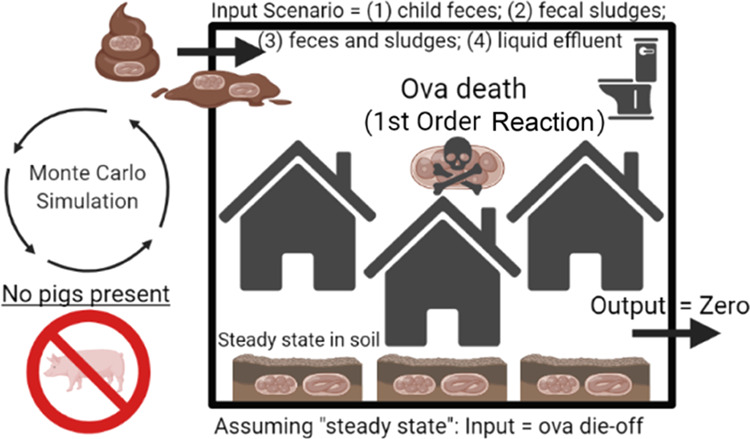

In low-income, urban, informal communities lacking sewerage and solid waste services, onsite sanitation (sludges, aqueous effluent) and child feces are potential sources of human fecal contamination in living environments. Working in informal communities of urban Maputo, Mozambique, we developed a quantitative, stochastic, mass-balance approach to evaluate plausible scenarios of localized contamination that could explain why the soil-transmitted helminth *Ascaris* remains endemic despite nearly universal coverage of latrines that sequester most fecal wastes. We used microscopy to enumerate presumptively viable *Ascaris* ova in feces, fecal sludges, and soils from compounds (i.e., household clusters) and then constructed a steady-state mass-balance model to evaluate possible contamination scenarios capable of explaining observed ova counts in soils. Observed *Ascaris* counts (mean = −0.01 log_10_ ova per wet gram of soil, sd = 0.71 log_10_) could be explained by deposits of 1.9 grams per day (10th percentile 0.04 grams, 90th percentile 84 grams) of child feces on average, rare fecal sludge contamination events that transport 17 kg every three years (10th percentile 1.0 kg, 90th percentile 260 kg), or a daily discharge of 2.7 kg aqueous effluent from an onsite system (10th percentile 0.09 kg, 90th percentile 82 kg). Results suggest that even limited intermittent flows of fecal wastes in this setting can result in a steady-state density of *Ascaris* ova in soils capable of sustaining transmission, given the high prevalence of *Ascaris* shedding by children (prevalence = 25%; mean = 3.7 log_10_ per wet gram, sd = 1.1 log_10_), the high *Ascaris* ova counts in fecal sludges (prevalence = 88%; mean = 1.8 log_10_ per wet gram, sd = 0.95 log_10_), and the extended persistence and viability of *Ascaris* ova in soils. Even near-universal coverage of onsite sanitation may allow for sustained transmission of *Ascaris* under these conditions.

## Abstract 2

A mass-balance approach to *Ascaris* transmission helps explain why improved sequestration of fecal waste is needed to protect public health in urban informal settlements.

## Background

When human feces is not safely managed, common in low- and middle-income countries,^1^ susceptible individuals may be exposed to enteric pathogens through well-understood pathways.^2^ The environmental persistence of a pathogen is dependent on its characteristics and a range of environmental conditions, including temperature, moisture content, and UV exposure.^3,4^ By accounting for enteric pathogens entering and leaving a defined system and their proliferation or inactivation over time, a mass-balance approach for estimating fecal waste and fecal pathogen flows in specific settings of interest is possible. Such an approach may yield insights into the suitability of localized control strategies (e.g., improved onsite sanitation, safe child and animal feces management) to reduce exposures. In communities where onsite sanitation predominates and fecal wastes are initially sequestered in latrine pits, septic tanks, or other containment structures, further transport of wastes and accompanying pathogens is possible via emptying/desludging, flooding, leakage, aqueous effluent discharge or via flies and cockroaches.^5−7^ Such flows are typically quantitatively minor in comparison with the mass of fecal waste effectively sequestered in onsite sanitation systems but may still result in exposure risks if the mobilized pathogens in these media maintain viability in sufficient numbers to infect new hosts. Fecal wastes can also be released directly into the environment via open defecation or improper disposal of child feces,^8,9^ which is possible even where sanitation coverage is good; the presence of and access to a latrine does not guarantee use by all members of a household all of the time.^10^ In addition, fecal wastes from animals may also contribute enteric pathogens to the living environment.^11,12^ Modeling the transport of human feces to soils may help explain why some onsite sanitation interventions did not achieve substantial reductions in enteric pathogen detection in soils^13,14^ and may help inform and prioritize intervention strategies to control exposures and reduce the risk of infection, disease, and sequelae associated with enteric pathogens.^15−18^

Recent cluster-randomized trials of sanitation interventions—and sanitation combined with water treatment and hygiene interventions—found mixed effects on child health outcomes. Some trials observed no effect on either child diarrhea or growth;^10,19−21^ Luby et al. found a reduction in diarrhea but not growth,^22^ while other trials observed improvements in growth but not diarrhea.^23,24^ These heterogeneous effects on child health outcomes may be because the interventions did not sufficiently reduce the transport of human and animal fecal wastes into the living environment.^22,25^

Soils are studied in the context of sanitation and health because they may act as an important environmental pathway for enteric pathogens. Numerous studies have observed widespread fecal contamination in soils collected in and around the living environment.^13,14,26^ The enteric pathogens present in these soils reflect circulating enteric pathogens from ineffectively contained animal^11,12^ and human wastes.^13,14^ Enteric microbes have often been cultured from soils, suggesting the potential for infectivity at the point of sampling.^27,28^ Soil ingestion then poses a risk of infection to infants and young children where and when viable pathogens are present from the living or play environment.^29^ Potential infection risks may be high^29^ for the pathogens (e.g., protozoa and helminths) that can maintain viability for extended periods in soils.^30,31^

Assessments of fecal contamination in soils often measure *Escherichia coli*, a fecal indicator bacterium, which can become naturalized in soils,^32^ or pathogen-associated nucleic acids via PCR. Quantitative estimates of the transport of child feces and fecal sludges to soils would be useful to inform sanitation interventions. However, using *E. coli* may overestimate the quantity transported due to the possibility of *E. coli* proliferation in soils. PCR-based approaches are feasible but would require consideration of nucleic acid persistence in soil.

Alternatively, the ova of *Ascaris*, a genus of soil-transmitted helminth (STH), can persist and maintain viability for years in soils,^33^ are only produced in the intestinal tract,^34^ and are commonly found in soils from endemic areas,^35−38^ and microscopic enumeration of ova is considered the gold standard.^35−39^ An estimated 760 million people worldwide^40^ are infected by *Ascaris*. Limitations to using *Ascaris* ova to estimate the transport of feces and sludges to soils include that ova are only shed by a subset of the population in endemic settings, microscopy requires highly trained technicians, and the ova of*Ascaris lumbricoides*, the species that infects humans, are morphologically similar to *Ascaris* ova shed by some animals (e.g., pigs shed*Ascaris suum*). Where these animals are absent, microscopic detection of *Ascaris* ova presents an opportunity to estimate fecal loading to soils.

Our research aim was to estimate the mass of human fecal loading to soils, bounded by confidence intervals, in four scenarios using a stochastic mass balance of *Ascaris* ova in soils from the living environment in Maputo, Mozambique. In scenario one, we assumed that all *Ascaris* ova enumerated in soils were transported from child feces; in scenario two, that all *Ascaris* ova were transported from fecal sludges; in scenario three, that *Ascaris* ova were transported from child feces and fecal sludges; and in scenario four, that all *Ascaris* ova were transported from the aqueous effluent of an onsite sanitation system. We subsequently modeled the transport of *Ascaris* ova to soils and quantitatively compared child feces, fecal sludges, and onsite sanitation system effluent as potential pathways of enteric pathogen transmission in this setting.

## Methods

### Study Setting

This study was situated within the Maputo Sanitation (MapSan) trial, a controlled before-and-after trial that evaluated the impact of an urban onsite sanitation intervention (i.e., a pour-flush toilet to a septic tank with a soakaway pit) on children’s health outcomes.^41^ The study was located in low-income, informal neighborhoods of Maputo, Mozambique, where population density exceeded 15,000 inhabitants per km^2^, sanitary conditions were poor, and the burden of disease was high.^41,42^ In this setting, clusters of households form compounds, which have a wall or fence to clearly delineate the property boundary. No pigs were present in the study area (Figure S1). The nongovernmental organization that delivered the sanitation intervention aimed to improve fecal sludge management in the study neighborhoods,^43^ but the intervention did not address child feces disposal practices. Knee *et al*. reported no impact of the onsite intervention on diarrhea or enteric pathogen carriage among intervention in children compared to those in the control group.^41^ At the 24-month follow-up of the MapSan trial, 5.6% (15/270) of intervention compounds and 30% (74/247) of control compounds reported emptying their onsite sanitation system in the previous 12 months, while 29% (289/980) of children aged one month to seven years defecated directly into the latrine.^41^ Among compounds that emptied in the previous year, most intervention compounds (10/15) reported mechanical emptying with a pump or vacuum truck, while most control compounds (67/74) reported manual emptying with buckets and shovels.^41^ While some residents were unsure where their fecal waste was ultimately disposed, most (57/74) control compounds and some intervention compounds (4/15) reported burying the pit contents inside the compound. The widespread detection of culturable *E. coli*(27) and pathogen genes^13^ in soils 24 months post-intervention suggests that the intervention did not sufficiently reduce exposures despite nearly exclusive use (97%) among households served.^41^

### Four Scenarios

We relied on several fundamental assumptions and sources of data to estimate the transport of feces, fecal sludges, and aqueous effluent to soils inside a hypothetical compound (Table S1). First, we assumed that the soils from the four sampling points per compound were representative of the soils in the localized area. In addition, we assumed that ova only entered the system from child feces, fecal sludges, or effluent, that no soil or ova leave the boundary of the system, and that soil ingestion by residents is negligible compared to the quantity of soil in the localized area. Further, we applied a steady-state assumption to the number of ova in the localized area. We applied these assumptions to four plausible scenarios.

**Figure 1:**
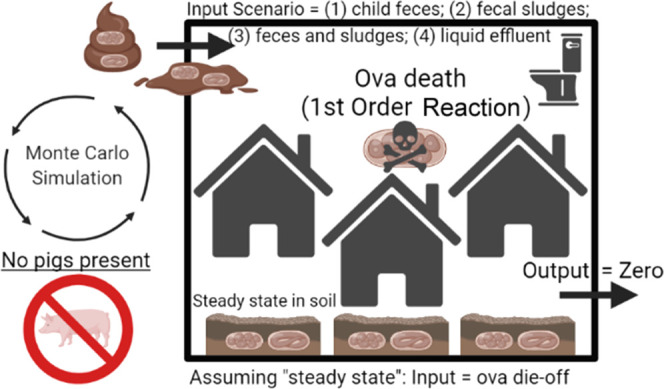
Model scenarios.

In scenario one, we assumed that the daily die-off of ova in the system was equal to the number of ova transported to soil from child feces (Figure 1). There was nearly universal latrine coverage and use during the MapSan trial, suggesting that the loading from adult open defecation may be negligible compared to the loading from child feces.^44^

In scenario two, we assumed that the total ova die-off across the 1095-day timeframe was equal to the number of ova transported from fecal sludge to soil during a single event (e.g., emptying, overflow during flooding, or leakage). While infants and younger children are more likely to use diapers or a child potty, older children are more likely to defecate directly into the latrine.^8,45^ It is therefore expected that in some compounds, all children defecate directly into the latrine and the transport of child feces to soils may be negligible.

In scenario three, we assumed that the daily number of ova transported from child feces to soil was equal to half the daily ova die-off, and the resulting difference in ova at day 1095 compared to the initial value was equal to the number of ova transported to soil during a single transport event on day 1095. We assumed this ratio to demonstrate the potential transport that occurs from both sources, but no evidence exists to justify this assumption as more likely than a different assumption.

In scenario four, we assumed that the daily die-off of ova in the system was equal to the number of ova transported to soil daily from the onsite sanitation system’s aqueous effluent. While we did not observe sanitation systems with direct discharge to soil in this setting,^27^ these systems are common in other low-income urban areas,^46−48^ and these systems plausibly existed in study neighborhoods.

### Mass-Balance Model

We applied a mass-balance approach to a hypothetical compound in Maputo, Mozambique. Presumptively viable *Ascaris* ova were modeled stochastically in soil, stool, fecal sludge, and effluent (eq 1) to model four scenarios (Text S1). We use Ova_in*,i*_ to denote the number of ova transported into the system on day *i*, Ova_out*,i*_ for the number of ova that leave the system, and −*r*_*a,i*_ to represent the number of ova that die off.
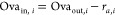
1

Assuming that Ova_out*,i*_ = 0, because the quantity of soil that is transported out of the living environment or is ingested by residents is negligible, compared to the total quantity of soil in the system, we can rearrange our equation to Ova_in*,i*_ = −*r*_*a,i*_. The quantity of ova transported into the system (Ova_in*,i*_) can be described as the product of the concentration of ova in child feces, fecal sludge, or aqueous effluent (*C*_ova*,i*_) and the mass (*m*_in,*i*_) (eq 2).
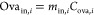
2

The die-off of ova (−*r*_*a,i*_) can be described by eq 3, where *N*_*t*,(*i*–1)_ is the initial number of ova in soil from the localized area and *N*_*t,i*_ is the number of ova remaining on day *i*.
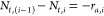
3

Rearranging these equations enables us to solve for *m*_in_, the mass of child feces or fecal sludge transported into the system (eq 4).
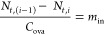
4

### Initial Number of Ova in Soils

We define the localized area for this study as the living environment for a hypothetical compound in a low-income urban unplanned settlement in Maputo, Mozambique (Text S2). We estimated the mass of soil that may potentially contain *Ascaris* ova by accounting for the median compound surface area (*SA* = 124 m^2^), the median percentage of the living environment covered by hardscape flooring (*P* = 74%), the depth to which *Ascaris* ova are likely transported (*d* = 0.5 cm), and the density of soil (ρ_soil_ = 1.7 g/cm^3^)^49^ (Text S2 and eq 5).
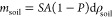
5

We used maximum likelihood estimation (MLE, *NADA* package in R) to fit a log-normal distribution to the observed number of viable ova in soils (Figure S2). In this process, we accounted for our recovery efficiency from sandy soil,^50^ which was 43% (Text S3). We randomly sampled the resulting log-normal distribution (Table S1) 274,000 times − the result of eq 5 − to stochastically assign each gram of soil in the system a quantity of viable *Ascaris* ova. We estimated *N*_*t*,(*i*–1)_ (eq 3) for day 0 by summing the ova count across the simulated 274,000 grams of soil inside the system boundary.

### Ova Die-Off

We assume a well-mixed batch reactor system that is recharged by feces, fecal sludge, or aqueous effluent, based on our simplifying assumptions. The die-off of *Ascaris* ova each day can then be described by first-order kinetics in a batch system (eq 6).^51^
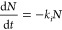
6

Assuming isothermal conditions (i.e., *k* is the constant) for each day, we integrate eq 6 to obtain eq 7.
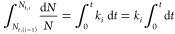
7

Integrating eq 7 then yields eq 8.
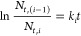
8

After rearranging eq 8, we can then solve for *N*_*t,i*_, the number of viable ova remaining in the system, using eq 9.
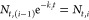
9

We calculated the decay constant, *k*, for each day using a temperature-dependent equation developed from the literature for wet soil with a pH of 7.2 (eq 10 and Figure S3).^31^ We obtained temperature data corresponding to the three years preceding sample collection (June 1, 2015 to May 31, 2018) from the National Oceanic and Atmospheric Administration (Global Historical Climatology Network Daily Summary, https://www.noaa.gov/). In rare instances (*n* = 16 days) where data were not available, we used the previous day’s temperature data. With this approach, each day was modeled independently, and the number of ova estimated to die-off (i.e., *N*_*t*,(*i*–1)_ – *N*_*t*,*i*_) each day was dependent on the historical average daily air temperature.
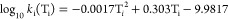
10

### Transport of Ova into the System

We modeled the transport of ova from child feces and liquid effluent to soil as a daily occurrence because both processes are likely to occur each day. However, we modeled the transport of ova in fecal sludge to soil after 1095 days (i.e., 3 years) because we previously observed that the mechanisms (e.g., emptying and flooding) that transport fecal sludge to the environment in this setting were infrequent.^43^ We selected 1095 days as the model timeframe because this represents the approximate amount of time from when MapSan trial data collection began and soil sampling occurred.^41^

### Soil Collection and Microscopy

In May 2018, we purposively collected soil at four locations from 90 compounds enrolled in the MapSan trial as part of a previous study.^27^ A subset of 140 samples from 35 compounds (15 intervention and 20 control compounds) were randomly selected via a random number generator for inclusion in this study. Standardized sample locations included a point 0.25 meters directly in front of (A) the household entrance, (B) the household’s solid waste storage container or pile, (C) the shared latrine entrance, and (D) a point where daily activities were frequently performed (e.g., dish or clothes washing and meal preparation). These locations were selected because pilot testing indicated they were easily identifiable by compound members and consistently accessible by field staff. Approximately 100 cm^3^ of soil was homogenized at each location and aliquoted into cryovials using an aluminum scoopula sterilized with 10% bleach and 70% ethanol between each sample. Samples were transported on ice to the Mozambican National Institute of Health (INS) in Maputo, Mozambique, where they were stored at −80 °C. All samples were shipped from Maputo, Mozambique, to Atlanta, GA, on dry ice (−80 °C) with temperature monitoring for analysis.

We developed and validated a rapid density flotation-based method to recover and enumerate helminth ova from soil (Text S4 and S5). Four grams of soil was combined with 10 mL of NaNO_3_ solution (specific gravity = 1.25) containing 0.1% Tween 80 in a sterile 15 mL centrifuge tube. The tube was shaken for two minutes and centrifuged at 500*g* for five minutes, and the resulting supernatant was analyzed using three mini-FLOTAC^52^ disks. Controlled experiments indicated recoveries of 43% from sandy soil, 16% from silty soil, and 77% from loamy soil (Text S3). In addition, we analyzed replicates from 20% of samples and used the mean of the two replicates as the overall result.

### Fecal Sludge Collection and Microscopy

From October 2017–April 2018, we collected fecal sludges from onsite sanitation systems at a subset of intervention and control compounds enrolled in the MapSan trial. We randomly selected 18 samples (nine intervention, nine control) for microscopy from those that had previously tested positive for *A. lumbricoides* via PCR (prevalence = 88%)^53^ because PCR is more sensitive than microscopy for helminth ova, and we accounted for nondetects with eq 11. Detailed sample collection methods are described elsewhere.^53^ Briefly, we used a sludge nabber (Nasco, Fort Atkinson, WI) to collect fecal sludge from the surface of pit latrines and a modified Wheaton subsurface sampler (Fisher Scientific, Waltham, MA) to collect fecal sludge from the surface of the solid portion inside septic tanks. Sampling devices were sterilized with 10% bleach and 70% ethanol between uses. Sludge was collected into sterile 50 mL centrifuge tubes, transported to INS on ice, aliquoted into cryovials, stored at −80 °C, and shipped to Atlanta, GA, on dry ice.

We adapted the mini-FLOTAC^52^ method for enumerating helminth ova from stools and soils for fecal sludges (Text S6). First, we added 0.5 grams of fecal sludge (wet weight) and 10 mL of NaNO_3_ solution (specific gravity = 1.25) into a sterile 15 mL centrifuge tube. Then, we manually shook the mixture for 20 seconds, pipetted 6 mL from the mixture to fill three mini-FLOTAC disks, waited for 10 minutes, rotated the disks, and then read the disks at 100× magnification.

### Stool Collection and Microscopy

We collected stool from children aged 1 to 72 months as part of the MapSan trial.^41^ Each enrolled child and their caregiver were provided with stool collection supplies, including diapers or a child potty for older children no longer using a diaper. Field workers returned the following day to collect the stool specimens, which were stored on ice and transported to the Mozambican National Institute of Health’s Parasitology Lab. On the same day as sample collection, a lab technician at the Parasitology Lab enumerated helminth ova using the single-slide Kato-Katz technique (Vestergaard Frandsen, Lausanne, Switzerland).^41^ The MapSan trial protocol was approved by the Comité Nacional de Bioética para a Saúde (CNBS), Ministério da Saúde (333/CNBS/14), the Research Ethics Committee of the London School of Hygiene & Tropical Medicine (reference # 8345), and the Institutional Review Board of the Georgia Institute of Technology (protocol # H15160).

### Ova Classification

As soils and fecal sludges were frozen for molecular analysis,^13,53^ we were unable to perform traditional STH viability assays after a period of embryonation.^54^ Instead, we used Schmitz *et al*.^55^ and other illustrative guides from the literature^56−59^ based on the lifecycle of *Ascaris* to classify ova as presumptively viable (Text S7). Any ova observed in the lifecycle of *Ascaris* from the single-cell stage to ova containing a visible larva were considered presumptively viable (hereafter referred to as viable ova). We used this approach because Cruz et al. indicated that early stages of ova development can further develop into infectious stages and should be considered when assessing viability.^59^ For a subset of soil samples and all fecal sludge samples, we also recorded the number of ova that appeared nonviable based on morphological characteristics (e.g., internal bubbling from heat inactivation)^57^ and the number that was infertile or dead.^55^ We assumed that all fertilized ova enumerated in stools were viable and infertile ova were not viable.

We accounted for child feces, fecal sludge, and aqueous effluent from which we did not detect viable *Ascaris* ova using eq 11. We divided each estimate of *m*_in_ for stools or fecal sludges and aqueous effluent by the percentage (*P*_a_) of children shedding *Ascaris* ova or pits containing ova, respectively.
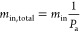
11

### Estimated Density of Ova in Stools and Fecal Sludges

We assumed that if a child’s stool did not test positive^41^ for any *Ascaris* ova, then that child did not shed *Ascaris* ova, and likewise, if a fecal sludge sample was not positive for *Ascaris* ova via PCR, then that onsite system did not contain *Ascaris* ova. Exclusively infertile *Ascaris* ova were observed in stool from a small subset of children. Due to the biological plausibility that these children also shed fertilized viable ova, we imputed a random value from one to the Kato-Katz LOD, 24 ova per gram, for these children. Then, we used MLE (*fitdistr* package in R) to fit a log-normal distribution to the observed concentrations of viable ova in stools and fecal sludges.^60^ We did not include nondetect data to fit distributions because this would have negatively impacted the distribution fit to the data. Instead, we accounted for the input of feces and fecal sludges that did not contain ova using eq 11. This approach produced log-normal distributions characterizing the *Ascaris* ova density in child feces and fecal sludges, which we used as an input to our mass-balance model.

In addition, we estimated that the concentration of ova in the effluent was 5.5% of the concentration observed in fecal sludge. This value is based on the ratio of total solid concentrations reported for sludge and effluent in Manga 2017.^61^

### Monte Carlo Simulation

To propagate the variability in *C*_ova_ for stools, fecal sludges, and aqueous effluent, we modeled eq 4 as a Monte Carlo simulation in R (version 4.0.4) (Figures S4 and S5). We randomly sampled from the log-normal distribution of ova in stools and aqueous effluent 10 times for each day, which we input to eq 4 to generate 10 different estimates of *m*_in_ per day. The simulation ran using data for 1095 days and generated a total of 10,950 daily estimates of *m*_in_ for each matrix. We modeled the transport of fecal sludges to soils as an event on day 1095. To propagate the variability in *C*_ova_ for fecal sludge, we randomly sampled from the distribution of ova in fecal sludge 10,950 times to retain similarity with stool. Then, we solved eq 4 for *m*_in_ using these estimates of *C*_ova_ and calculated *m*_in,total_ using eq 11. Finally, we pooled the daily estimates for stools and aqueous effluent across the entire timeframe and the estimates for fecal sludges from day 1095 to generate summary statistics.

### Sensitivity Analysis

We conducted a sensitivity analysis by running the Monte Carlo simulation using different assumptions, including soil depth, soil density, recovery efficiency, fecal sludge transport frequency, and viability, to evaluate how changes in model parameters would impact our point estimates.

## Results

### Soils

**Figure 2:**
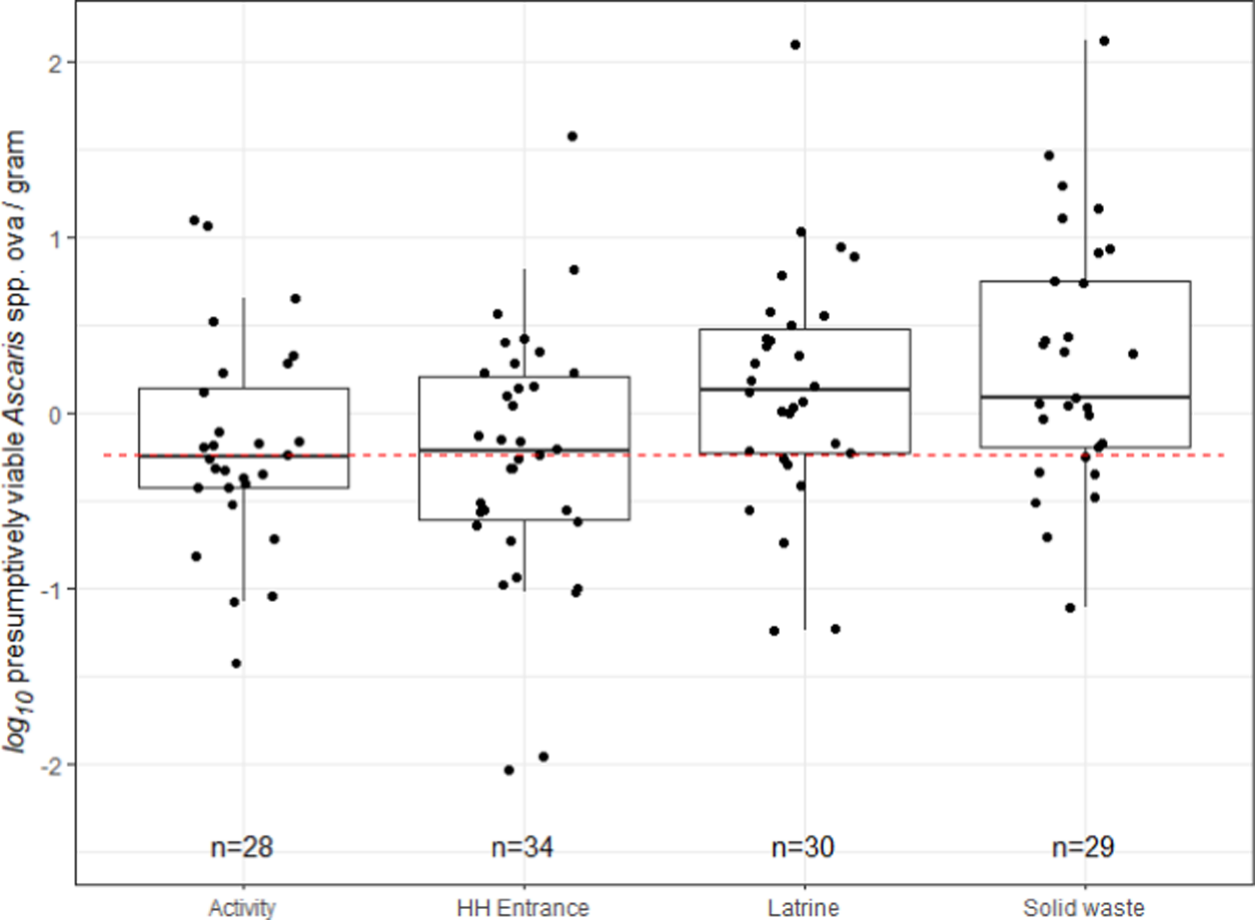
Presumptively viable *Ascaris* ova in soil samples by compound location (empirical data). Nondetects were imputed below the LOD, which is shown as the dashed red line.

We observed ≥1 viable *Ascaris* ova in 64% (78/121) of soil samples, with a mean of −0.01 log_10_ (sd = 0.71 log_10_) viable ova per gram of wet soil and a median of 1.3 ova per gram (Table 1). The viable ova counts per gram of wet soil were higher at latrine entrances (median = 1.4) and solid waste storage areas (median = 1.2), compared to household entrances (median = 0.61) and activity areas (median = nondetect) (Figure 2 and Table S2). We excluded 19 samples from analysis because they were evaluated using an incorrect concentration of Tween 80, while 121 samples were included. The median difference among the 27 replicates assessed was 0.67 viable ova per gram wet soil with an intraclass correlation coefficient (ICC) of 0.80, indicating good reliability.^62^ Among the 21 replicates analyzed by different technicians, the ICC was 0.76.

**Table 1:** *Ascaris* Ova in Soils, Fecal sludges, and Child Feces

*Ascaris* classification	≥1 ova observed	mean (SD)	median (IQR)
Soil (*Ascaris* ova per gram wet)			
presumptively viable	64% (78/121)	–0.01 log_10_ (0.71 log_10_)	1.3 (ND, 2.5)
presumptively nonviable	60% (54/90)	–0.35 log_10_ (0.84 log_10_)	0.66 (ND, 1.7)
any ova	78% (70/90)	0.45 log_10_ (0.69 log_10_)	3.8 (1.2, 7.7)
Fecal Sludge (*Ascaris* ova per gram wet)			
presumptively viable	100% (18/18)	1.8 log_10_ (0.95 log_10_)	41 (8, 310)
presumptively nonviable	100% (18/18)	1.3 log_10_ (1.1 log_10_)	19 (3.5, 57)
any ova	100% (18/18)	2.1 log_10_ (0.88 log_10_)	87 (29, 430)
Child Feces (*Ascaris* ova per gram wet)			
presumptively viable ova	23% (124/545)^a^	3.7 log_10_ (1.1 log_10_)	8400 (1500, 25000)

a Stools (2%) were positive exclusively for infertile *Ascaris* ova. The concentrations of presumptively viable ova for these samples were imputed from 1 ovum per gram to the LOD of 24. ND = nondetect.

### Fecal Sludges

We observed ≥1 viable *Ascaris* ova in all fecal sludge samples (*n* = 18) that had been randomly chosen among those previously positive by PCR. The mean number of viable *Ascaris* ova per gram of fecal sludge (wet weight) was 1.8 log_10_ (sd = 0.95 log_10_), and the median was 41 ova per gram of fecal sludge (Figure S6).

### Child Feces

We observed fertile *Ascaris* ova in 23% (124/545) of child feces and exclusively infertile *Ascaris* ova in 1.8% (10/545) of child feces. Among children shedding ova, there was a mean of 3.7 log_10_ (sd = 1.1 log_10_) ova per gram of feces and a median of 8,400 ova per gram of feces (Figure S6).

### Estimated Mass Transported to Achieve Steady State

For model scenario one, where we assumed that all *Ascaris* ova in soil were transported from child feces, we estimated that the 10th percentile of fresh child feces transported to soil per day in the localized area was 0.04 grams, the 50th percentile was 1.9 grams, and the 90th percentile was 84 grams (Table 2). For model scenario two, where we assumed that all *Ascaris* ova were transported from fecal sludge to nearby soils during a single triennial event, we estimated that the 10th percentile of fecal sludge transported to soil was 1000 grams, the 50th percentile was 17,000 grams, and the 90th percentile was 260,000 grams. For scenario three, we assumed that half the daily die-off of *Ascaris* ova in soil was replaced by child feces, and fecal sludge transported *Ascaris* ova equivalent to the ova die-off after three years. In this scenario, we estimated that the 50th percentile of daily child feces transported to soil was 0.13 grams and the 50th percentile of annual fecal sludge transported to soil was 17,000 grams. For model scenario four, where we assumed that all *Ascaris* ova were transported from aqueous effluent, we estimated that the 10th percentile of effluent transported to soil per day was 91 grams, the 50th percentile was 2700 grams, and the 90th percentile was 82,000 grams (Table 2).

**Table 2:** Estimates of Child Feces and Fecal Sludges Transported to Soil

	grams transported to soil per day			grams transported to soil during a single event on day 1095		
	percentile of model output					
transport scenario	10th	50th	90th	10th	50th	90th
1. only child feces	0.04	1.9	84	NA	NA	NA
2. only fecal sludge	NA	NA	NA	1000	17,000	260,000
3. both child feces and fecal sludge	2.4 × 10^–3^	0.13	7.8	1100	17,000	260,000
4. only aqueous effluent	91	2700	82,000	NA	NA	NA

### Sensitivity Analysis

Increasing the soil depth, soil density, recovery efficiency, and viable *Ascaris* ova in soil, which are used to calculate the initial number of viable ova in soils (i.e., *N*_*t*,0_), resulted in increased mass loading estimates (Table S3). In scenario two, transport frequency had little effect on the estimated fecal sludge loading because nearly all *Ascaris* died off in the first year (Figure S7). Likewise, in scenario three, most of ova that died off needed to be replaced by ova from child feces to meaningfully reduce the estimated loading of fecal sludge during a transport event on day 1095 (Table S3).

## Discussion

Viable *Ascaris* ova were prevalent in soils and fecal sludges from household living environments in low-income urban communities in Maputo, Mozambique, with nearly universal onsite sanitation coverage. The observed concentration of *Ascaris* ova in soil could be held at the steady state by a relatively small amount of child feces or aqueous effluent transported to soil daily or fecal sludge transported triennially. These findings suggest that nearly universal coverage of onsite sanitation alone may be insufficient to control pathogens such as *Ascaris* in endemic settings like Maputo.

The fact that each of these scenarios represents quantitatively limited inputs of fecal material reveals a key insight from this work: in endemic settings where STH and other enteric infections are common, the stakes for effective operation of sanitation infrastructure are high. Low- and middle-income settings are typically where basic technologies like pit latrines are proposed as solutions to achieve public health goals of sanitation expansion, but at the same time, they are places where even minor fecal flows carry non-negligible risks to people who may come into contact with environmental media (e.g., soils) contaminated with fecal wastes. The apparent challenge for the water, sanitation, and hygiene (WASH) sector and for sanitation innovation specifically is to develop control strategies that are completely effective in removing excreta from downstream human contact. Such strategies may require a substantial change from previous paradigms in WASH innovation, representing “transformative” approaches to controlling exposures.^63−65^ The definition of transformative WASH is debated, and while sewer systems remain a long-term goal in low-income settings, consensus^63^ is forming that packages of interventions tailored to locally relevant sources of fecal contamination, in addition to universal coverage of onsite sanitation, may be the foundation of transformative WASH.

The relative importance of the transmission routes we evaluated may vary in different contexts based on local sanitation infrastructure and practices. Additional work to define the localized relevance of these transmission routes, including in rural and peri-urban areas, would be helpful to inform interventions. Further, our model was limited to soil in the localized area. Exposures to *Ascaris* ova and other enteric pathogens occur through multiple well-understood pathways.^2^ Universal, comprehensive sanitation coverage requires safe management at each step in the disposal chain to reduce exposures.^66^

The concept of a threshold effect, meaning that health gains are realized after a certain threshold of community level sanitation coverage is achieved, is important to the WASH field.^67^ Identification of these tipping points has been an aim of several studies to elucidate the hypothesized relationship with health outcomes.^68−70^ Our results directly inform this type of work. If such a threshold exists for *Ascaris*, and potentially other enteric pathogens, it may require universal onsite sanitation coverage, as well as other complementary interventions that interrupt transmission from child feces, fecal sludges, and liquid effluent.

In scenario two, we estimated that the vast majority of *Ascaris* ova died off in the first year, which has been demonstrated empirically under similar environmental conditions to those in Maputo.^3,71,72^ This provides further evidence that infrequent emptying is preferred over frequent emptying, given safe sequestration in the onsite system. Infrequent emptying limits the potential for spills, leaks, and aerosolization of sludge.^73^ During the 24-month phase of the MapSan trial, only 5.6% of intervention compounds reported emptying in the previous year, and most did so using hygienic mechanized emptying.^43^ On the other hand, 30% of control compounds emptied in the previous year, and most used less hygienic manual emptying. This difference may have contributed to the 38% reduction in the prevalence of *A. lumbricoides* DNA that we observed in intervention latrine entrance soils compared to controls during the 24-month follow-up.^13^

Any defecation event that does not completely and directly dispose of feces into an onsite containment structure or a toilet connected to a sewage network poses a risk of transporting some feces to the environment. Open defecation likely transfers some feces to soil even if the stool is picked up later, disposable diapers can break open or leak while stored in a solid waste pile, and reusable diapers require washing in water, which may be dumped onto nearby soil.^8,9,74^ This real-world heterogeneity, ranging from entire discarded stools to small quantities of stool transported from diapers or wash water, is reflected in the wide confidence interval around our child feces loading estimate in scenario one.

The age at which children begin directly using latrines varies based on prevalent sanitation technologies, culture, and other contextual determinants. A study in rural and peri-urban Cambodia found the mean age when caregivers believed their child could independently use a latrine was five years old.^75^ In low-income informal settlements in India, the median age caregivers reported beginning latrine training was three years and that they expected independent latrine use at five years. At the 24-month follow-up of the MapSan trial, only 29% (289/980) of children, who were one month to seven years old, defecated directly into the latrine.^41^ After this initial defecation event, MapSan trial caregivers reported that 17% (37/224) of child feces was ultimately disposed of in a latrine from children under two years and that 95% (547/574) was disposed in a latrine from children over two. In rural Bangladesh, caregivers reported that 89% of children under three years and 40% of children aged three to eight practiced open defecation.^76^ Open defecation by children may occur because caregivers believe that latrine use by their child would be unsafe or that their child is not developmentally capable.^8^ Latrine training mats, which offer increased safety and accessibility, are one potential intervention to increase latrine use by developmentally capable children.^77^

There are limitations to our data collection and laboratory methods. First, we assessed *presumptive* viability, which potentially misclassified ova. We estimated that 50% of *Ascaris* ova in soils were viable, which is less than a recent study in rural Kenya (99%) and Bangladesh (70%).^78^ It is possible that we misclassified some roundworm ova from other animals, such as *Toxocara* from cats and dogs or *Ascaridia galli* from poultry, as *Ascaris* ova. However, this was unlikely because *Toxocara* are morphologically distinct, and we found no difference in ova counts between compounds with and without these animals (Figures S8 and SS9). In addition, we collected fecal sludges near the surface of the solids in pit latrines and septic tanks, which represents relatively fresh feces. Ova at greater depths may have experienced greater die-off, suggesting that our methods underestimated the transport of fecal sludges to soils.

In addition, there are limitations to the modeling structure and parameters used. First, we applied simplifying assumptions to samples collected cross-sectionally, which permitted steady-state conditions, but included historical temperature data to account for temporal variation. We calculated the decay constant, *k*, via air temperature, not soil temperature, and assumed a constant pH and that the soil was wet. Variation in moisture content, pH, and sunlight may have resulted in an underestimation of *Ascaris* ova die-off and subsequently underestimated the mass transported to soils. We also assumed a closed system, but mechanisms such as walking, wind, and yard cleaning can transport ova into or out of the localized area. Further, we only used data from children ≤7 years. However, there were likely no large-scale sources of *Ascaris* ova except for child feces. Adult residents reported nearly universal latrine use and no pigs were present which eliminated the possibility of zoonotic shedding (e.g., of *A. suum*(41)). We observed greater *Ascaris* ova counts in soils from latrine entrances and solid waste storage areas compared to household entrances and activity areas. By including these locations where ova may be more likely, we may have overestimated the initial number of ova in the system and subsequently overestimated fecal mass loading. Finally, this analysis represents a single pathogen in one low-income setting and required endemicity of *Ascaris*.^41,79^ Yet, the flexibility of this approach offers the opportunity for similar mass-balance approaches using other common enteric pathogens that do not reproduce outside the gut (i.e., viruses, protozoa, and helminths) and targets shed universally in feces (e.g., human mitochondrial DNA^80^). Such models may advance our understanding of how fecal wastes are transported to the localized environment.

At a localized scale in a low-income urban community, we estimated that a relatively small quantity of child feces or aqueous effluent transported daily to soil or a moderate quantity of fecal sludge transported infrequently could plausibly explain the observed density of *Ascaris* ova in soils. In highly endemic settings, this indicates that nearly all fecal wastes must be safely sequestered because even small releases to the environment could allow the cycle of infection to continue. Foundational to helminth control efforts is mass drug administration (MDA), but in endemic settings, MDA is a short-term treatment strategy that should be accompanied by improvements to sanitation, hygiene, and housing to break the cycle of infection.^81,82^ However, onsite sanitation interventions have not demonstrated substantial reductions in environmental fecal contamination,^13,83,84^ and this work suggests that even nearly universal coverage of these systems alone may be insufficient to interrupt *Ascaris* transmission in endemic settings. Instead, a sustainable environmental response to the risks posed by helminths and other fecal-oral pathogens will require policies and strategies capable of achieving a nearly complete reduction in the child feces and fecal sludges transported to the living environment.

## Notes

All data used in this paper will be available at a dedicated data repository at Open Science Framework (OSF.io) upon acceptance. Permanent link: https://osf.io/8r4ty/.

## Supporting Information Available

Text S1. Model framing; Text S2. mass of soil containing *Ascaris*; Text S3. recovery experiments; Text S4. microscopy training; Text S5. soil protocol; Figure S1. MapSan trial area; Figure S2. empirical and simulated ova in soil; Text S6. fecal sludge microscopy protocol; Text S7. presumptive *Ascaris* viability assessment; Figure S3. equation for decay constant *k*; Figure S4. empirical and simulated ova in fecal sludges; Figure S5. empirical and simulated ova in stool; Table S1. model parameters; Table S2. comparison of ova counts in soil; Figure S6. *Ascaris* concentration by matrix; Figure S7. ova in the system over time; Table S3. sensitivity analysis; Figure S8. chicken or ducks present; and Figure S9. dogs or cats present (PDF)

## References

[ref1] World Health Organization. Progress on Household Drinking Water, Sanitation and Hygiene 2000–2017. Special Focus on Inequalities; World Health Organization, 2019.

[ref2] Wagner E.; Lanoix J. Excreta Disposal for Rural Areas and Small Communities. Monogr Ser World Heal. Organ. 1958, 39, 1–182. 13581743

[ref3] Brown H. W. Studies on the Rate of Development and Viability of the Eggs of *Ascaris Lumbricoides* and Trichuris Trichiura under Field Conditions. J. Parasitol. 1927, 14, 1–15. 10.2307/3271397.

[ref4] Rose J.; Jiménez-Cisneros B.; Murphy H. Persistence of Pathogens in Sewage and Other Water Types. In Global Water Pathogen Project; Michigan State University, 2019 10.14321/waterpathogens.51.

[ref5] Knee J.; Sumner T.; Adriano Z.; Anderson C.; Capone D.; Eng B.; Casmo V.; Holcomb D.; Macdougall A.; Molotkova E.; Monteiro Braga J.; Russo C.; Peter Schmidt W.; Stewart J.; Zambrana W.; Zuin V.; Nalá R.; Cumming O.; Brown J. Effects of an Urban Sanitation Intervention on Childhood Enteric Infection and Diarrhoea in Mozambique medRxiv 2020, 10.1101/2020.08.20.20178608. PMC8121544 33835026

[ref6] Adenusi A. A.; Akinyemi M. I.; Akinsanya D. Domiciliary Cockroaches as Carriers of Human Intestinal Parasites in Lagos Metropolis, Southwest Nigeria: Implications for Public Health. J. Arthropod. Borne. Dis. 2018, 12, 141–151. 10.18502/jad.v12i2.40. 30123808 PMC6091797

[ref7] Greenberg B. Flies and Disease Volume II: Biology and Disease Transmission, 1st ed.; Princeton University Press: Princeton, NJ, 1973.

[ref8] Bauza V.; Majorin F.; Routray P.; Sclar G. D.; Caruso B. A.; Clasen T. Child Feces Management Practices and Fecal Contamination: A Cross-Sectional Study in Rural Odisha, India. Sci. Total Environ. 2020, 709, 136169 10.1016/j.scitotenv.2019.136169. 31905545 PMC7031693

[ref9] Bauza V.; Madadi V.; Ocharo R. M.; Nguyen T. H.; Guest J. S. Microbial Source Tracking Using 16S RRNA Amplicon Sequencing Identifies Evidence of Widespread Contamination from Young Children’s Feces in an Urban Slum of Nairobi, Kenya. Environ. Sci. Technol. 2019, 53, 8271–8281. 10.1021/acs.est.8b06583. 31268313

[ref10] Clasen T.; Boisson S.; Routray P.; Torondel B.; Bell M.; Cumming O.; Ensink J.; Freeman M.; Jenkins M.; Odagiri M.; Ray S.; Sinha A.; Suar M.; Schmidt W. P. Effectiveness of a Rural Sanitation Programme on Diarrhoea, Soil-Transmitted Helminth Infection, and Child Malnutrition in Odisha, India: A Cluster-Randomised Trial. Lancet Global Health 2014, 2, e645–e653. 10.1016/S2214-109X(14)70307-9. 25442689

[ref11] Berendes D. M.; Yang P. J.; Lai A.; Hu D.; Brown J. Estimation of Global Recoverable Human and Animal Faecal Biomass. Nat. Sustainability 2018, 1, 679–685. 10.1038/s41893-018-0167-0. PMC10922008 38464867

[ref12] Penakalapati G.; Swarthout J.; Delahoy M. J.; McAliley L.; Wodnik B.; Levy K.; Freeman M. C. Exposure to Animal Feces and Human Health: A Systematic Review and Proposed Research Priorities. Environ. Sci. Technol. 2017, 51, 11537–11552. 10.1021/acs.est.7b02811. 28926696 PMC5647569

[ref13] Capone D.; Berendes D.; Cumming O.; Holcomb D.; Knee J.; Konstantinidis K. T.; Levy K.; Nalá R.; Risk B. B.; Stewart J.; Brown J. Impact of an Urban Sanitation Intervention on Enteric Pathogen Detection in Soils. Environ. Sci. Technol. 2021, 55, 9989–10000. 10.1021/acs.est.1c02168. 34236178 PMC8327413

[ref14] Fuhrmeister E. R.; Ercumen A.; Pickering A. J.; Jeanis K. M.; Crider Y.; Ahmed M.; Brown S.; Alam M.; Sen D.; Islam S.; Kabir M. H.; Islam M.; Rahman M.; Kwong L. H.; Arnold B. F.; Luby S. P.; Colford J. M.; Nelson K. L. Effect of Sanitation Improvements on Pathogens and Microbial Source Tracking Markers in the Rural Bangladeshi Household Environment. Environ. Sci. Technol. 2020, 54, 4316–4326. 10.1021/acs.est.9b04835. 32167305 PMC7144219

[ref15] Humphrey J. H. Child Undernutrition, Tropical Enteropathy, Toilets, and Handwashing. Lancet 374, 1032 1035. 10.1016/S0140-6736(09)60950-8. 19766883

[ref16] Platts-Mills J. A.; Liu J.; Rogawski E. T.; Kabir F.; Lertsethtakarn P.; Siguas M.; Khan S. S.; Praharaj I.; Murei A.; Nshama R.; Mujaga B.; Havt A.; Maciel I. A.; McMurry T. L.; Operario D. J.; Taniuchi M.; Gratz J.; Stroup S. E.; Roberts J. H.; Kalam A.; Aziz F.; Qureshi S.; Islam M. O.; Sakpaisal P.; Silapong S.; Yori P. P.; Rajendiran R.; Benny B.; McGrath M.; McCormick B. J. J.; Seidman J. C.; Lang D.; Gottlieb M.; Guerrant R. L.; Lima A. A. M.; Leite J. P.; Samie A.; Bessong P. O.; Page N.; Bodhidatta L.; Mason C.; Shrestha S.; Kiwelu I.; Mduma E. R.; Iqbal N. T.; Bhutta Z. A.; Ahmed T.; Haque R.; Kang G.; Kosek M. N.; Houpt E. R.; Acosta A. M.; Rios de Burga R.; Chavez C. B.; Flores J. T.; Olotegui M. P.; Pinedo S. R.; Trigoso D. R.; Vasquez A. O.; Ahmed I.; Alam D.; Ali A.; Rasheed M.; Soofi S.; Turab A.; Yousafzai A.; Zaidi A. K.; Shrestha B.; Rayamajhi B. B.; Strand T.; Ammu G.; Babji S.; Bose A.; George A. T.; Hariraju D.; Jennifer M. S.; John S.; Kaki S.; Karunakaran P.; Koshy B.; Lazarus R. P.; Muliyil J.; Ragasudha P.; Raghava M. V.; Raju S.; Ramachandran A.; Ramadas R.; Ramanujam K.; Rose A.; Roshan R.; Sharma S. L.; Sundaram S.; Thomas R. J.; Pan W. K.; Ambikapathi R.; Carreon J. D.; Doan V.; Hoest C.; Knobler S.; Miller M. A.; Psaki S.; Rasmussen Z.; Richard S. A.; Tountas K. H.; Svensen E.; Amour C.; Bayyo E.; Mvungi R.; Pascal J.; Yarrot L.; Barrett L.; Dillingham R.; Petri W. A.; Scharf R.; Ahmed A. S.; Alam M. A.; Haque U.; Hossain M. I.; Islam M.; Mahfuz M.; Mondal D.; Nahar B.; Tofail F.; Chandyo R. K.; Shrestha P. S.; Shrestha R.; Ulak M.; Bauck A.; Black R.; Caulfield L.; Checkley W.; Lee G.; Schulze K.; Scott S.; Murray-Kolb L. E.; Ross A. C.; Schaefer B.; Simons S.; Pendergast L.; Abreu C. B.; Costa H.; Di Moura A.; Filho J. Q.; Leite Á. M.; Lima N. L.; Lima I. F.; Maciel B. L.; Medeiros P. H.; Moraes M.; Mota F. S.; Oriá R. B.; Quetz J.; Soares A. M.; Mota R. M.; Patil C. L.; Mahopo C.; Maphula A.; Nyathi E. Use of Quantitative Molecular Diagnostic Methods to Assess the Aetiology, Burden, and Clinical Characteristics of Diarrhoea in Children in Low-Resource Settings: A Reanalysis of the MAL-ED Cohort Study. Lancet Global Health 2018, 6, e1309–e1318. 10.1016/S2214-109X(18)30349-8. 30287127 PMC6227251

[ref17] Prendergast A. J.; Kelly P. Interactions between Intestinal Pathogens, Enteropathy and Malnutrition in Developing Countries. Curr. Opin. Infect. Dis. 2016, 29, 229–236. 10.1097/QCO.0000000000000261. 26967147 PMC4888918

[ref18] Kosek M. N.; Ahmed T.; Bhutta Z.; Caulfield L.; Guerrant R.; Houpt E.; Kang G.; Kosek M.; Lee G.; Lima A.; McCormick B. J. J.; Platts-Mills J.; Seidman J.; Caulfield L.; Kosek M.; Lee G.; McCormick B. J. J.; Seidman J.; Blank R. R.; Gottlieb M.; Knobler S. L.; Lang D. R.; Miller M. A.; Tountas K. H.; Bhutta Z. A.; Caulfield L.; Checkley W.; Guerrant R. L.; Houpt E.; Kosek M. N.; Lang D. R.; Mason C. J.; Miller M. A.; Murray-Kolb L. E.; Petri W. A.; Seidman J. C.; Ahmed T.; Bessong P.; Bhutta Z. A.; Haque R.; John S.; Kang G.; Kosek M. N.; Lima A. A. M.; Mduma E. R.; Oriá R. B.; Shrestha P. S.; Shrestha S. K.; Svensen E.; Zaidi A. K. M.; Abreu C. B.; Acosta A. M.; Ahmed I.; Shamsir Ahmed A. M.; Ali A.; Ambikapathi R.; Barrett L.; Bauck A.; Bayyo E.; Bodhidatta L.; Bose A.; Daniel Carreon J.; Chandyo R. K.; Charu V.; Costa H.; Dillingham R.; Di Moura A.; Doan V.; Filho J. Q.; Graham J.; Hoest C.; Hossain I.; Islam M.; Steffi Jennifer M.; Kaki S.; Koshy B.; Lee G.; Leite Á. M.; Lima N. L.; Maciel B. L. L.; Mahfuz M.; Mahopo C.; Maphula A.; McCormick B. J. J.; McGrath M.; Mohale A.; Moraes M.; Mota F. S.; Muliyil J.; Mvungi R.; Nayyar G.; Nyathi E.; Olortegui M. P.; Oria R.; Vasquez A. O.; Pan W. K.; Pascal J.; Patil C. L.; Pendergast L.; Pinedo S. R.; Platts-Mills J.; Psaki S.; Raghava M. V.; Ramanujam K.; Rasheed M.; Rasmussen Z. A.; Richard S. A.; Rose A.; Roshan R.; Schaefer B.; Scharf R.; Seidman J. C.; Sharma S. L.; Shrestha B.; Shrestha R.; Simons S.; Soares A. M.; Mota R. M. S.; Soofi S.; Strand T.; Tofail F.; Thomas R. J.; Turab A.; Ulak M.; Wang V.; Yarrot L.; Yori P. P.; Alam D.; Ambikapathi R.; Amour C.; Chavez C. B.; Babji S.; de Burga R. R.; Doan V.; Flores J. T.; Gratz J.; George A. T.; Hariraju D.; Havt A.; Houpt E.; Karunakaran P.; Lazarus R. P.; Lima I. F.; McGrath M.; Mondal D.; Medeiros P. H. Q. S.; Nshama R.; Quetz J.; Qureshi S.; Raju S.; Ramachandran A.; Ramadas R.; Catharine Ross A.; Salas M. S.; Samie A.; Schulze K.; Seidman J. C.; Shanmuga Sundaram E.; Swema B. M.; Trigoso D. R. Causal Pathways from Enteropathogens to Environmental Enteropathy: Findings from the MAL-ED Birth Cohort Study. EBioMedicine 2017, 18, 109–117. 10.1016/j.ebiom.2017.02.024. 28396264 PMC5405169

[ref19] Arnold B. F.; Khush R. S.; Ramaswamy P.; London A. G.; Rajkumar P.; Ramaprabha P.; Durairaj N.; Hubbard A. E.; Balakrishnan K.; Colford J. M. Causal Inference Methods to Study Nonrandomized, Preexisting Development Interventions. Proc. Natl. Acad. Sci. U.S.A. 2010, 107, 22605–22610. 10.1073/pnas.1008944107. 21149699 PMC3012527

[ref20] Null C.; Stewart C. P.; Pickering A. J.; Dentz H. N.; Arnold B. F.; Arnold C. D.; Benjamin-Chung J.; Clasen T.; Dewey K. G.; Fernald L. C. H.; Hubbard A. E.; Kariger P.; Lin A.; Luby S. P.; Mertens A.; Njenga S. M.; Nyambane G.; Ram P. K.; Colford J. M. Effects of Water Quality, Sanitation, Handwashing, and Nutritional Interventions on Diarrhoea and Child Growth in Rural Kenya: A Cluster-Randomised Controlled Trial. Lancet. Global Health 2018, 6, e316–e329. 10.1016/S2214-109X(18)30005-6. 29396219 PMC5809717

[ref21] Patil S. R.; Arnold B. F.; Salvatore A. L.; Briceno B.; Ganguly S.; Colford J. M.; Gertler P. J. The Effect of India’s Total Sanitation Campaign on Defecation Behaviors and Child Health in Rural Madhya Pradesh: A Cluster Randomized Controlled Trial. PLoS Med. 2014, 11, e1001709 10.1371/journal.pmed.1001709. 25157929 PMC4144850

[ref22] Luby S. P.; Rahman M.; Arnold B. F.; Unicomb L.; Ashraf S.; Winch P. J.; Stewart C. P.; Begum F.; Hussain F.; Benjamin-Chung J.; Leontsini E.; Naser A. M.; Parvez S. M.; Hubbard A. E.; Lin A.; Nizame F. A.; Jannat K.; Ercumen A.; Ram P. K.; Das K. K.; Abedin J.; Clasen T. F.; Dewey K. G.; Fernald L. C.; Null C.; Ahmed T.; Colford J. M. Effects of Water Quality, Sanitation, Handwashing, and Nutritional Interventions on Diarrhoea and Child Growth in Rural Bangladesh: A Cluster Randomised Controlled Trial. Lancet Global Health 2018, 6, e302–e315. 10.1016/S2214-109X(17)30490-4. 29396217 PMC5809718

[ref23] Pickering A. J.; Djebbari H.; Lopez C.; Coulibaly M.; Alzua M. L. Effect of a Community-Led Sanitation Intervention on Child Diarrhoea and Child Growth in Rural Mali: A Cluster-Randomised Controlled Trial. Lancet Global Health 2015, 3, e701–11. 10.1016/S2214-109X(15)00144-8. 26475017

[ref24] Reese H.; Routray P.; Torondel B.; Sinharoy S. S.; Mishra S.; Freeman M. C.; Chang H. H.; Clasen T. Assessing Longer-Term Effectiveness of a Combined Household-Level Piped Water and Sanitation Intervention on Child Diarrhoea, Acute Respiratory Infection, Soil-Transmitted Helminth Infection and Nutritional Status: A Matched Cohort Study in Rural Odisha. Int. J. Epidemiol. 2019, 48, 1757–1767. 10.1093/ije/dyz157. 31363748 PMC6929523

[ref25] Pickering A. J.; Swarthout J.; Mureithi M.; Mboya J.; Arnold B. F.; Wolfe M.; Dentz H. N.; Lin A.; Arnold C. D.; Rao G.; Stewart C. P.; Ram P. K.; Clasen T.; Colford J. M.; Null C. Can Individual and Integrated Water, Sanitation, and Handwashing Interventions Reduce Fecal Contamination in the Household Environment? Evidence from the WASH Benefits Cluster-Randomized Trial in Rural Kenya. bioRxiv 2019, 731992 10.1101/731992.

[ref26] Baker K. K.; Senesac R.; Sewell D.; Sen Gupta A.; Cumming O.; Mumma J. Fecal Fingerprints of Enteric Pathogen Contamination in Public Environments of Kisumu, Kenya Associated with Human Sanitation Conditions and Domestic Animals. Environ. Sci. Technol. 2018, 52, 10263–10274. 10.1021/acs.est.8b01528. 30106283 PMC6557411

[ref27] Capone D.; Adriano Z.; Berendes D.; Cumming O.; Dreibelbis R.; Holcomb D. A.; Knee J.; Ross I.; Brown J. A Localized Sanitation Status Index as a Proxy for Fecal Contamination in Urban Maputo, Mozambique. PLoS One 2019, 14, e0224333 10.1371/journal.pone.0224333. 31652287 PMC6814227

[ref28] Navab-Daneshmand T.; Friedrich M. N. D.; Gächter M.; Montealegre M. C.; Mlambo L. S.; Nhiwatiwa T.; Mosler H. J.; Julian T. R. *Escherichia Coli* Contamination across Multiple Environmental Compartments (Soil, Hands, Drinking Water, and Handwashing Water) in Urban Harare: Correlations and Risk Factors. Am. J. Trop. Med. Hyg. 2018, 98, 803–813. 10.4269/ajtmh.17-0521. 29363444 PMC5930891

[ref29] Capone D.; Bivins A.; Knee J.; Cumming O.; Nalá R.; Brown J. Quantitative Microbial Risk Assessment of Pediatric Infections Attributable to Ingestion of Fecally Contaminated Domestic Soils in Low-Income Urban Maputo, Mozambique. Environ. Sci. Technol. 2021, 55, 1941–1952. 10.1021/acs.est.0c06972. 33472364 PMC7860170

[ref30] Olson M. E.; Goh J.; Phillips M.; Guselle N.; McAllister T. A. Giardia Cyst and Cryptosporidium Oocyst Survival in Water, Soil, and Cattle Feces. J. Environ. Qual. 1999, 28, 1991 10.2134/jeq.1999.00472425002800060040x.

[ref31] Senecal J.; Nordin A.; Vinnerås B. Fate of Ascaris at Various PH, Temperature and Moisture Levels. J. Water Health 2020, 18, 375–382. 10.2166/wh.2020.264. 32589622

[ref32] Ishii S.; Ksoll W. B.; Hicks R. E.; Sadowsky M. J. Presence and Growth of Naturalized *Escherichia Coli* in Temperate Soils from Lake Superior Watersheds. Appl. Environ. Microbiol. 2006, 72, 612–621. 10.1128/AEM.72.1.612-621.2006. 16391098 PMC1352292

[ref33] Asaolu S. O.; Ofoezie I. E. Ascaris Spp. In Global Water Pathogen Project; Robertson L., Ed.; Michigan State University, 2019 10.14321/waterpathogens.41.

[ref34] Holland C. Ascaris: The Neglected Parasite, 1st ed.; Elsevier Inc: London, UK, 2013.

[ref35] Steinbaum L.; Njenga S. M.; Kihara J.; Boehm A. B.; Davis J.; Null C.; Pickering A. J. Soil-Transmitted Helminth Eggs Are Present in Soil at Multiple Locations within Households in Rural Kenya. PLoS One 2016, 11, e0157780 10.1371/journal.pone.0157780. 27341102 PMC4920396

[ref36] Schulz S.; Kroeger A. Soil Contamination with *Ascaris Lumbricoides* Eggs as an Indicator of Environmental Hygiene in Urban Areas of North-East Brazil. J. Trop. Med. Hyg. 1992, 95, 95–103. 1560490

[ref37] Muller M.; Sánchez R. M.; Suswillo R. R. Evaluation of a Sanitation Programme Using Eggs of *Ascaris Lumbricoides* in Household Yard Soils as Indicators. J. Trop. Med. Hyg. 1989, 92, 10–16. 2918573

[ref38] Kumwenda S.; Msefula C.; Kadewa W.; Diness Y.; Kato C.; Morse T.; Ngwira B. Is There a Difference in Prevalence of Helminths between Households Using Ecological Sanitation and Those Using Traditional Pit Latrines? A Latrine Based Cross Sectional Comparative Study in Malawi. BMC Res. Notes 2017, 10, 200 10.1186/s13104-017-2519-7. 28599671 PMC5466731

[ref39] Organization W. H. Ending the Neglect to Attain the Sustainable Development Goals: A Road Map for Neglected Tropical Diseases 2021–2030: Overview, 2020.

[ref40] Brooker S. J.; Pullan R. L. Ascaris *Lumbricoides and Ascariasis*: Estimating Numbers Infected and Burden of Disease. In Ascaris: The Neglected Parasite; Elsevier, 2013; pp 343–362 10.1016/B978-0-12-396978-1.00013-6.

[ref41] Knee J.; Sumner T.; Adriano Z.; Anderson C.; Bush F.; Capone D.; Casmo V.; Holcomb D. A.; Kolsky P.; MacDougall A.; Molotkova E.; Braga J. M.; Russo C.; Schmidt W. P.; Stewart J.; Zambrana W.; Zuin V.; Nalá R.; Cumming O.; Brown J. Effects of an Urban Sanitation Intervention on Childhood Enteric Infection and Diarrhea in Maputo, Mozambique: A Controlled before-and-after Trial. eLife 2021, 10, e62278 10.7554/eLife.62278. 33835026 PMC8121544

[ref42] WaterAid. Spatial Planning for Urban Sanitation and Water, Maputo, 2013.

[ref43] Capone D.; Buxton H.; Cumming O.; Dreibelbis R.; Knee J.; Nalá R.; Ross I.; Brown J. Impact of an Intervention to Improve Pit Latrine Emptying Practices in Low Income Urban Neighborhoods of Maputo, Mozambique. Int. J. Hyg. Environ. Health 2020, 226, 113480 10.1016/j.ijheh.2020.113480. 32086016 PMC7184672

[ref44] Bick S.; Buxton H.; Chase R. P.; Ross I.; Adriano Z.; Capone D.; Knee J.; Brown J.; Nalá R.; Cumming O.; Dreibelbis R. Using Path Analysis to Test Theory of Change: A Quantitative Process Evaluation of the MapSan Trial. BMC Public Health 2021, 21, 1411 10.1186/s12889-021-11364-w. 34271913 PMC8285873

[ref45] Knee J.; Sumner T.; Adriano Z.; Berendes D.; de Bruijn E.; Schmidt W.-P.; Nalá R.; Cumming O.; Brown J. Risk Factors for Childhood Enteric Infection in Urban Maputo, Mozambique: A Cross-Sectional Study. PLoS Negl. Trop. Dis. 2018, 12, e0006956 10.1371/journal.pntd.0006956. 30419034 PMC6258421

[ref46] Berendes D. M.; de Mondesert L.; Kirby A. E.; Yakubu H.; Adomako L.; Michiel J.; Raj S.; Robb K.; Wang Y.; Doe B.; Ampofo J.; Moe C. L. Variation in *E. Coli* Concentrations in Open Drains across Neighborhoods in Accra, Ghana: The Influence of Onsite Sanitation Coverage and Interconnectedness of Urban Environments. Int. J. Hyg. Environ. Health 2020, 224, 113433 10.1016/j.ijheh.2019.113433. 31978730 PMC6996153

[ref47] Berendes D. M.; Leon J. S.; Kirby A. E.; Clennon J. A.; Raj S. J.; Yakubu H.; Robb K. A.; Kartikeyan A.; Hemavathy P.; Gunasekaran A.; Roy S.; Ghale B. C.; Kumar J. S.; Mohan V. R.; Kang G.; Moe C. L. Associations between Open Drain Flooding and Pediatric Enteric Infections in the MAL-ED Cohort in a Low-Income, Urban Neighborhood in Vellore, India. BMC Public Health 2019, 19, 926 10.1186/s12889-019-7268-1. 31291914 PMC6617624

[ref48] Foster T.; Falletta J.; Amin N.; Rahman M.; Liu P.; Raj S.; Mills F.; Petterson S.; Norman G.; Moe C.; Willetts J. Modelling Faecal Pathogen Flows and Health Risks in Urban Bangladesh: Implications for Sanitation Decision Making. Int. J. Hyg. Environ. Health 2021, 233, 113669 10.1016/j.ijheh.2020.113669. 33578186

[ref49] USDA Natural Resources Conservation Service. Soil Quality Indicators. https://www.nrcs.usda.gov. (Accessed Jun 25, 2021).

[ref50] Vicente E. M.; Jermy C. A.; Schreiner H. D. In Urban Geology of Maputo, Mocambique; 10th Congress of the International Association for Engineering Geology and the Environment; London Geological Society: London, 2006.

[ref51] von Sperling M.; Verbyla M. E.; Mihelcic J. Understanding Pathogen Reduction in Sanitation Systems: Units of Measurement, Expressing Changes in Concentrations, and Kinetics. In Water and Sanitation for the 21st Century: Health and Microbiological Aspects of Excreta and Wastewater Management (Global Water Pathogen Project); Verbyla M. E.; Mihelcic J. R., Eds.; Michigan State University, 2019 10.14321/waterpathogens.54.

[ref52] Cringoli G.; Maurelli M. P.; Levecke B.; Bosco A.; Vercruysse J.; Utzinger J.; Rinaldi L. The Mini-FLOTAC Technique for the Diagnosis of Helminth and Protozoan Infections in Humans and Animals. Nat. Protoc. 2017, 12, 1723–1732. 10.1038/nprot.2017.067. 28771238

[ref53] Capone D.; Berendes D.; Cumming O.; Knee J.; Nalá R.; Risk B. B.; Stauber C.; Zhu K.; Brown J. Analysis of Fecal Sludges Reveals Common Enteric Pathogens in Urban Maputo, Mozambique. Environ. Sci. Technol. Lett. 2020, 7, 889–895. 10.1021/acs.estlett.0c00610. PMC11177333 38881628

[ref54] Collender P. A.; Kirby A. E.; Addiss D. G.; Freeman M. C.; Remais J. V. Methods for Quantification of Soil-Transmitted Helminths in Environmental Media: Current Techniques and Recent Advances. Trends Parasitol. 2015, 31, 625–639. 10.1016/j.pt.2015.08.007. 26440788 PMC4679500

[ref55] Schmitz B.; Pearce-Walker J.; Gerba C.; Pepper I. A Method for Determining Ascaris Viability Based on Early-to-Late Stage In-Vitro Ova Development. J. Residuals Sci. Technol. 2016, 13, 275–286. 10.12783/issn.1544-8053/13/4/5.

[ref56] US EPA: National Risk Management Research Laboratory. Environmental Regulations and Technology: Control of Pathogens and Vector Attraction in Sewage Sludge, Cincinnati, OH, 2003.

[ref57] Butkus M. A.; Hughes K. T.; Bowman D. D.; Liotta J. L.; Jenkins M. B.; Labare M. P. Inactivation of *Ascaris Suum* by Short-Chain Fatty Acids. Appl. Environ. Microbiol. 2011, 77, 363–366. 10.1128/AEM.01675-10. 21057018 PMC3019692

[ref58] Kim M.-K.; Pyo K.-H.; Hwang Y.-S.; Park K. H.; Hwang I. G.; Chai J.-Y.; Shin E.-H. Effect of Temperature on Embryonation of *Ascaris Suum* Eggs in an Environmental Chamber. Korean J. Parasitol. 2012, 50, 239–242. 10.3347/kjp.2012.50.3.239. 22949753 PMC3428571

[ref59] Cruz L. M.; Allanson M.; Kwa B.; Azizan A.; Izurieta R. Morphological Changes of Ascaris Spp. Eggs During Their Development Outside the Host. J. Parasitol. 2012, 98, 63–68. 10.1645/GE-2821.1. 21801007

[ref60] Delignette-Muller M. L.; Dutang C. Fitdistrplus: An R Package for Fitting Distributions. J. Stat. Software 2015, 64, 1–34. 10.18637/jss.v064.i04.

[ref61] Manga M. The Feasibility of Co-Composting as an Upscale Treatment Method for Fecal Sludge in Urban. Doctoral dissertation, Africa, University of Leeds: United Kingdom, 2017.

[ref62] Koo T. K.; Li M. Y. A Guideline of Selecting and Reporting Intraclass Correlation Coefficients for Reliability Research. J. Chiropr. Med. 2016, 15, 155 10.1016/J.JCM.2016.02.012. 27330520 PMC4913118

[ref63] Cumming O.; Arnold B. F.; Ban R.; Clasen T.; Esteves Mills J.; Freeman M. C.; Gordon B.; Guiteras R.; Howard G.; Hunter P. R.; Johnston R. B.; Pickering A. J.; Prendergast A. J.; Prüss-Ustün A.; Rosenboom J. W.; Spears D.; Sundberg S.; Wolf J.; Null C.; Luby S. P.; Humphrey J. H.; Colford J. M. The Implications of Three Major New Trials for the Effect of Water, Sanitation and Hygiene on Childhood Diarrhea and Stunting: A Consensus Statement. BMC Med. 2019, 17, 173 10.1186/s12916-019-1410-x. 31462230 PMC6712663

[ref64] Pickering A. J.; Null C.; Winch P. J.; Mangwadu G.; Arnold B. F.; Prendergast A. J.; Njenga S. M.; Rahman M.; Ntozini R.; Benjamin-Chung J.; Stewart C. P.; Huda T. M. N.; Moulton L. H.; Colford J. M.; Luby S. P.; Humphrey J. H. The WASH Benefits and SHINE Trials: Interpretation of WASH Intervention Effects on Linear Growth and Diarrhoea. Lancet Global Health 2019, 7, e1139–e1146. 10.1016/S2214-109X(19)30268-2. 31303300

[ref65] Husseini M.; Darboe M. K.; Moore S. E.; Nabwera H. M.; Prentice A. M. Thresholds of Socio-Economic and Environmental Conditions Necessary to Escape from Childhood Malnutrition: A Natural Experiment in Rural Gambia. BMC Med. 2018, 16, 199 10.1186/s12916-018-1179-3. 30382849 PMC6211595

[ref66] Berendes D. M.; Sumner T. A.; Brown J. M. Safely Managed Sanitation for All Means Fecal Sludge Management for at Least 1.8 Billion People in Low and Middle Income Countries. Environ. Sci. Technol. 2017, 51, 3074–3083. 10.1021/acs.est.6b06019. 28128924

[ref67] Wolf J.; Johnston R. B.; Hunter P. R.; Gordon B.; Medlicott K.; Prüss-Üstün A. A Faecal Contamination Index for Interpreting Heterogeneous Diarrhoea Impacts of Water, Sanitation and Hygiene Interventions and Overall, Regional and Country Estimates of Community Sanitation Coverage with a Focus on Low- and Middle-Income Countries. Int. J. Hyg. Environ. Health 2019, 222, 270–282. 10.1016/j.ijheh.2018.11.005. 30503228 PMC6417992

[ref68] Fuller J. A.; Villamor E.; Cevallos W.; Trostle J.; Eisenberg J. N. I Get Height with a Little Help from My Friends: Herd Protection from Sanitation on Child Growth in Rural Ecuador. Int. J. Epidemiol. 2016, 45, 460–469. 10.1093/ije/dyv368. 26936912 PMC5841884

[ref69] Garn J. V.; Boisson S.; Willis R.; Bakhtiari A.; Al-Khatib T.; Amer K.; Batcho W.; Courtright P.; Dejene M.; Goepogui A.; Kalua K.; Kebede B.; Macleod C. K.; Madeleine K.; Ii M.; Mbofana M. S. A.; Mpyet C.; Ndjemba J.; Olobio N.; Pavluck A. L.; Sokana O.; Southisombath K.; Taleo F.; Solomon A. W.; Freeman M. C. Sanitation and Water Supply Coverage Thresholds Associated with Active Trachoma: Modeling Cross-Sectional Data from 13 Countries. PLoS Negl. Trop. Dis. 2018, 12, e0006110 10.1371/journal.pntd.0006110. 29357365 PMC5800679

[ref70] Contreras J. D.; Islam M.; Mertens A.; Pickering A. J.; Kwong L. H.; Arnold B. F.; Benjamin-Chung J.; Hubbard A. E.; Alam M.; Sen D.; Islam S.; Rahman M.; Unicomb L.; Luby S. P.; Colford J. M.; Ercumen A. Influence of Community-Level Sanitation Coverage and Population Density on Environmental Fecal Contamination and Child Health in a Longitudinal Cohort in Rural Bangladesh. Int. J. Hyg. Environ. Health 2022, 245, 114031 10.1016/j.ijheh.2022.114031. 36058111 PMC9489923

[ref71] Jensen P. K.; Phuc P. D.; Konradsen F.; Klank L. T.; Dalsgaard A. Survival of Ascaris Eggs and Hygienic Quality of Human Excreta in Vietnamese Composting Latrines. Environ. Heal. 2009, 8, 57 10.1186/1476-069X-8-57. PMC2804663 20003550

[ref72] Mizgajska H. The Distribution and Survival of Eggs of *Ascaris Suum* in Six Different Natural Soil Profiles. Acta Parasitol. 1993, 4, 170–174.

[ref73] Farling S.; Rogers T.; Knee J. S.; Tilley E. A.; Brown J.; Deshusses M. A. Bioaerosol Emissions Associated with Pit Latrine Emptying Operations. Sci. Total Environ. 2019, 648, 1082–1086. 10.1016/j.scitotenv.2018.08.147. 30340254 PMC6234106

[ref74] Majorin F.; Torondel B.; Ka Seen Chan G.; Clasen T. Interventions to Improve Disposal of Child Faeces for Preventing Diarrhoea and Soil-Transmitted Helminth Infection. Cochrane Database Syst. Rev. 2019, 10.1002/14651858.CD011055.pub2. PMC6757260 31549742

[ref75] Miller-Petrie M. K.; Voigt L.; McLennan L.; Cairncross S.; Jenkins M. W. Infant and Young Child Feces Management and Enabling Products for Their Hygienic Collection, Transport, and Disposal in Cambodia. Am. J. Trop. Med. Hyg. 2016, 94, 456–465. 10.4269/ajtmh.15-0423. 26598568 PMC4751965

[ref76] Contreras J. D.; Islam M.; Mertens A.; Pickering A. J.; Kwong L. H.; Arnold B. F.; Benjamin-Chung J.; Hubbard A. E.; Alam M.; Sen D.; Islam S.; Rahman M.; Unicomb L.; Luby S. P.; Colford J. M.; Ercumen A. Longitudinal Effects of a Sanitation Intervention on Environmental Fecal Contamination in a Cluster-Randomized Controlled Trial in Rural Bangladesh. Environ. Sci. Technol. 2021, 55, 8169–8179. 10.1021/acs.est.1c01114. 34086447 PMC8213058

[ref77] Huda T. M. N.; Jahir T.; Sarker S.; Yeasmin F.; Masud A. Al.; Sultana J.; Das J. B.; Nizame F. A.; Leontsini E.; Shoab A. K.; Kwong L. H.; Rahman M.; Luby S. P.; Winch P. J. Formative Research to Design a Child-Friendly Latrine in Bangladesh. Int. J. Environ. Res. Public Health 2021, 18, 11092 10.3390/ijerph182111092. 34769612 PMC8583528

[ref78] Steinbaum L.; Kwong L. H.; Ercumen A.; Negash M. S.; Lovely A. J.; Njenga S. M.; Boehm A. B.; Pickering A. J.; Nelson K. L. Detecting and Enumerating Soil-Transmitted Helminth Eggs in Soil: New Method Development and Results from Field Testing in Kenya and Bangladesh. PLoS Negl. Trop. Dis. 2017, 11, e0005522 10.1371/journal.pntd.0005522. 28379956 PMC5393894

[ref79] Augusto G.; Nalá R.; Casmo V.; Sabonete A.; Mapaco L.; Monteiro J. Geographic Distribution and Prevalence of Schistosomiasis and Soil-Transmitted Helminths among Schoolchildren in Mozambique. Am. J. Trop. Med. Hyg. 2009, 81, 799–803. 10.4269/ajtmh.2009.08-0344. 19861614

[ref80] Zhu K.; Suttner B.; Pickering A.; Konstantinidis K. T.; Brown J. A Novel Droplet Digital PCR Human MtDNA Assay for Fecal Source Tracking. Water Res. 2020, 183, 116085 10.1016/j.watres.2020.116085. 32750535 PMC7495096

[ref81] Remais J. V.; Eisenberg J. N. Balance between Clinical and Environmental Responses to Infectious Diseases. Lancet 2012, 379, 1457–1459. 10.1016/S0140-6736(11)61227-0. 22475491 PMC3890320

[ref82] Mahmoud A.; Zerhouni E. Neglected Tropical Diseases: Moving Beyond Mass Drug Treatment To Understanding The Science. Health Aff. 2009, 28, 1726–1733. 10.1377/hlthaff.28.6.1726. 19887413

[ref83] Kwong L. H.; Sen D.; Islam S.; Shahriar S.; Benjamin-Chung J.; Arnold B. F.; Hubbard A.; Parvez S. M.; Unicomb L.; Rahman M.; Nelson K.; Colford J. M.; Luby S. P.; Ercumen A. Effect of Sanitation Improvements on Soil-Transmitted Helminth Eggs in Courtyard Soil from Rural Bangladesh: Evidence from a Cluster-Randomized Controlled Trial. bioRxiv 2020, 2020.09.29.318097. 10.1101/2020.09.29.318097. PMC8351931 34319986

[ref84] Steinbaum L.; Mboya J.; Mahoney R.; Njenga S. M.; Null C.; Pickering A. J. Effect of a Sanitation Intervention on Soil-Transmitted Helminth Prevalence and Concentration in Household Soil: A Cluster-Randomized Controlled Trial and Risk Factor Analysis. PLoS Negl. Trop. Dis. 2019, 13, e0007180 10.1371/journal.pntd.0007180. 30742614 PMC6386409

