# Supplementary material for: Persistent Ascaris Transmission Is Possible in Urban Areas Even Where Sanitation Coverage Is High
Source: Environ Sci Technol. 2022 Oct 26;56(22):15969–80. doi: 10.1021/acs.est.2c04667 (PMC9671051; doi:10.1021/acs.est.2c04667)
Supplement: Supplementary file 1 — es2c04667_si_001.pdf [file es2c04667_si_001.pdf]

Manuscript Title: Persistent *Ascaris* Transmission is Possible in Urban Areas Even Where Sanitation Coverage is High

Authors: Drew Capone[1], Troy Barker[2], Oliver Cumming[3], Abeoseh Flemister[4], Riley Geason[4], Elizabeth Kim[4], Jackie Knee[3], Yarrow Linden[2], Musa Manga[2], Mackenzie Meldrum[5], Rassul Nala[6], Simrill Smith[5], Joe Brown[2*]

1. Department of Environmental and Occupational Health, School of Public Health, Indiana University, Bloomington, Indiana, 47401, USA
2. Department of Environmental Sciences and Engineering, Gillings School of Public Health, University of North Carolina at Chapel Hill, Chapel Hill, North Carolina, 27599, USA
3. Department of Disease Control, London School of Hygiene and Tropical Medicine, London, WC1E 7HT, United Kingdom
4. Department of Biology, University of North Carolina at Chapel Hill, Chapel Hill, North Carolina, 27599, USA
5. Department of Civil and Environmental Engineering, Georgia Institute of Technology, Atlanta, Georgia, 30332, USA
6. Ministério da Saúde, Instituto Nacional de Saúde Maputo, Maputo, Mozambique

*Corresponding Author: Joe Brown, Phone: +1 919-360-8752, joebrown@unc.edu, Address: 135 Dauer Dr, Chapel Hill, North Carolina, 27599, USA

Supporting Information

1. Text S1. Model framing
2. Text S2. Mass of soil containing *Ascaris*
3. Text S3. Recovery Experiments
4. Text S4. Microscopy training
5. Text S5. Soil Protocol
6. Figure S1. MapSan Trial Area
7. Figure S2. Empirical and simulated ova in soil
8. Text S6. Fecal Sludge Microscopy Protocol
9. Text S7. Presumptive *Ascaris* Viability Assessment
10. Figure S3. Equation for Decay Constant *k*
11. Figure S4. Empirical and simulated ova in fecal sludges
12. Figure S5. Empirical and simulated ova in stool
13. Table S1. Model parameters
14. Table S2. Comparison of ova counts in soil
15. Figure S6. *Ascaris* concentration by matrix
16. Figure S7. Ova in the system over time
17. Table S3. Sensitivity analysis
18. Figure S8. Chicken or ducks present
19. Figure S9. Dogs or cats present

Text S1. Model framing

We used a mechanistic model to evaluate potential transport scenarios of *Ascaris* ova from child feces and fecal sludges to soils. There is evidence for household clustering of *Ascaris* infection.[1] We chose, however, to model a single hypothetical compound, instead of separate models from compounds with and without children shedding *Ascaris* ova for a number of reasons. First, we did not expect any difference in the quantity of child feces transported to soil at compounds with and without children infected by *Ascaris*. Next, we previously found that 88% of fecal sludge samples from MapSan trial tested positive *Ascaris lumbricoides* DNA, which suggests that other individuals besides the children who were enrolled in the trial may have been infected by *Ascaris* and would have been shedding *Ascaris* ova into the onsite sanitation system. Finally, there was a 1-13 month gap between when stool samples were collected (04/2017-04/2018) and when the soil samples were collected (05/2018). Given this gap, and the observed temporal heterogeneity in *Ascaris* infection (see tables below), we limited our approach to a single mechanistic model and did not attempt to account for household clustering in the model.

Temporal variation in *Ascaris* shedding among matched compounds.

≥1 child's stool tested positive for *Ascaris* in the compound at the 12-month follow-up	≥1 child’s stool tested positive for *Ascaris* in the matched compound at the 24-month follow-up	Prevalence
Yes	Yes	8.3% (30/360)
Yes	No	19% (68/360)
No	Yes	16% (56/360)
No	No	57% (205/360)

Temporal variation in *Ascaris* shedding among matched children.

Child’s stool tested positive for *Ascaris* at the 12-month follow-up	Matched child’s stool tested positive for *Ascaris* at the 24-month follow-up	Prevalence
Yes	Yes	11% (22/208)
Yes	No	18% (37/208)
No	Yes	17% (36/208)
No	No	54% (113/208)

Text S2. Mass of soil containing *Ascaris*

Enumerators visited compounds enrolled in the MapSan trial and asked the compound leader to indicate the compound boundary, which was recorded using GeoODK (GeoMarvel, Alexandria, Virginia). From a random subset of these compound polygons (n=69), we used the measure tool in ArcMap 10.8.1 (ESRI, Redlands, California) to determine the surface area covered by the roofs of buildings, as a proxy for finished floors, because 94% (759/805) of enrolled households had a covered floor[2]. The median compound surface area (SA) was 124 square meters (min = 31, max = 530) and we estimated 74% of this living environment (min = 25%, max = 98%) was covered by housing or pavement (P), resulting in a median soil surface area of 32 m^2 which may contain *Ascaris* ova. In addition, we assumed that ova were only transported to a depth of 0.5 cm (d) because evidence suggests soil-transmitted helminth ova are predominantly located at the surface[3] and there was a drought during the preceding three years[4] that limited the potential for ova to be transported into the subsurface[5]. Assuming the average density of soil (ρ_{soil}) is 1.7 g/cm^3, we calculated the total mass of soil in the localized area was 274 kilograms (Equation 5)[6].

Text S3. Recovery Experiments

We collected approximately 100 grams of sandy, silty, and loamy soil from the campus of the University of North Carolina at Chapel Hill in Chapel Hill, North Carolina. In a sterile 500 mL beaker we combined increments of 6-7 grams of each soil with 250 µL of fecal sludge containing 1,300 *Ascaris lumbricoides* ova per mL, pausing after each addition to homogenize the soil and fecal sludge for 30 seconds using a sterile wooden tongue depressor. This process resulted in a total mass of 25 grams of soil containing 1,300 *Ascaris lumbricoides* ova, which equates to 52 ova per gram wet soil. We placed the three soil samples in the fridge at 4°C overnight. The following day we used the methods describe in Text S1 to enumerate the ova. We performed our methods in duplicate.

**Trial #**	**1**	**2**	**Average**
Sand	46%	41%	43%
Silt	15%	17%	16%
Loam	72%	82%	77%

Limit of Detection Calculation

We analyzed approximately 4 grams of soil per sample, which indicates our theoretical limit of detection (LOD) was 0.25 ova/gram wet soil. As soils in Maputo are typically a sandy loam – Vincente *et al*. 2006 observed that soils were 71-96% sand – we used our recovery from sandy soil to calculate our experimentally determined LOD. Dividing the theoretical limit of detection (0.25) by our recovery efficiency (0.43), yield 0.58 ova/gram wet soil.

Text S4. Microscopy training

Stool samples were analyzed by the Mozambican National Institute of Health's Parasitology Lab, which has years of experience analyzing stool samples for helminth ova. Author DC first received training on helminth identification and enumeration from staff at the Parasitology Lab in Maputo, Mozambique. Second, Author DC received training in mini-FLOTAC and helminth identification and enumeration from the Kaplan Lab at the University of Georgia, which also serves as the US Distributor for mini-FLOTAC.

All lab technicians were trained by DC in helminth identification and enumeration for a separate study of helminth prevalence in children, except for author TB who previously worked for two years as a technician in a Veterinary Parasitology Lab.

Lab technicians were trained over a period 2-4 weeks. First, technicians spent one to two days reading the World Health Organization's "Bench Aid for the Diagnosis of Intestinal Parasites" (1st and 2nd editions), CDC DPDx's "Diagnostic Procedures for Intestinal Parasites" (https://www.cdc.gov/dpdx/diagnosticprocedures/stool/morphcomp.html), CDC DPDx's "Artifact Identification Sheet" (https://www.cdc.gov/dpdx/artifacts/index.html), Donald L. Price's "Procedure Manual for the Diagnosis of Intestinal Parasites"[7], and Ash and Orihel's "Human Parasitic Diseases: A Diagnostic Atlas"[8]. Then, technicians were trained for one day on using a microscope and practiced identifying ova that were fixed and mounted onto pre-prepared slides (VWR, Radnor, PA).

The study team then acquired and trained on mini-FLOTAC with feces from dogs, cats, chickens, pigs, horses, and cows that contained a wide range of helminth ova (e.g., hookworm, *Ascaris*, *Trichuris*, *Toxocara*, strongyle, and pinworm) and artifacts (e.g., pollen, undigested food, fungal spores, and mite eggs). Frozen human stool samples collected as part of the MapSan Trial[2] that contained *Ascaris* ova were used to train technicians to differentiate between viable and non-viable *Ascaris* ova. As the final step in training, lab technicians had to demonstrate the ability to enumerate ova within 25% of the count observed by DC. During the analysis phase of the study, technicians consulted with DC and TB for help classifying ova if they were uncertain. After training as completed the study team intermittently analyzed animal feces with and without ova to continue training. These analyses served as microscopy process controls.

Text S5. Soil Protocol

1. Tare a sterile 15 mL centrifuge tube
2. Mass 4.0 grams of soil into the tube (3.9 – 4.1 is acceptable)

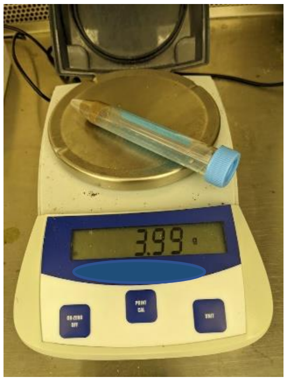

3. Add 10 mL of $NaNO_3$ (SG = 1.25) containing 0.1% Tween 80 to the 15 mL tube

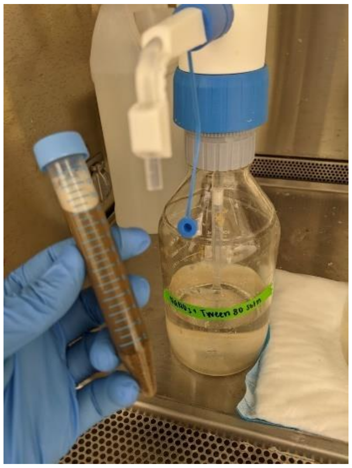

4. Manually shake the tube for two minutes

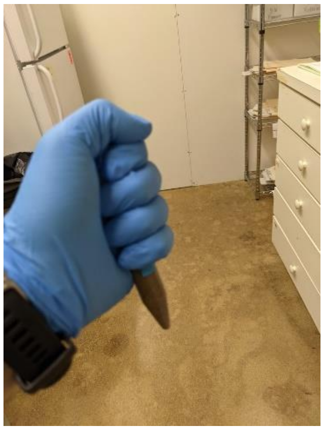

5. Centrifuge at 500xg for five minutes

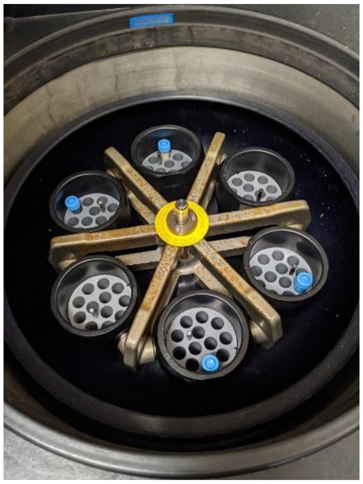

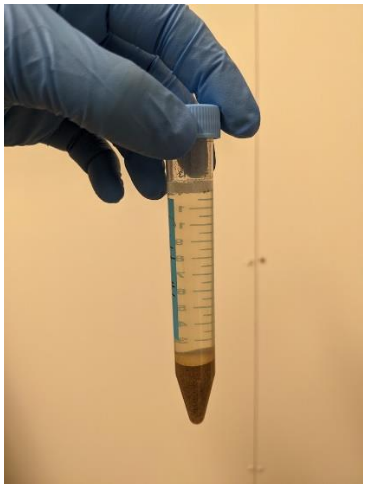

6. Pipet from the surface of the supernatant to fill three mini-FLOTAC discs
    a. See Cringoli *et al*. 2010 and Cringoli *et al*. 2017 for additional information on the mini-FLOTAC technique

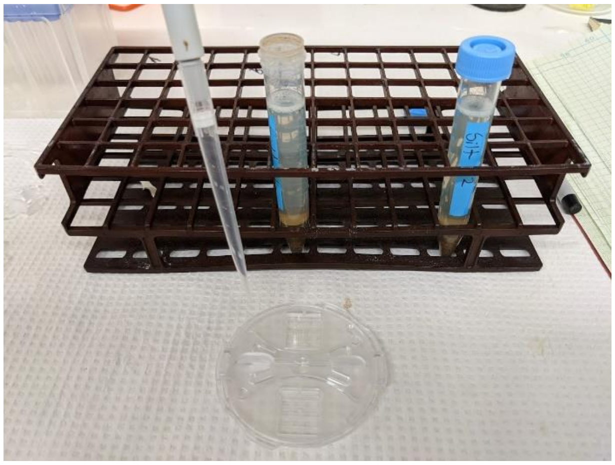

7. Wait 10 minutes, then turn each disc using the attached key

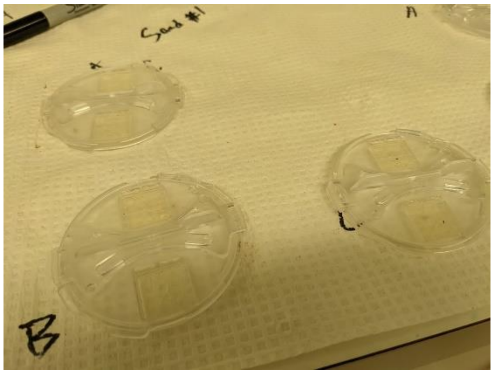

8. Read the discs at 100X magnification

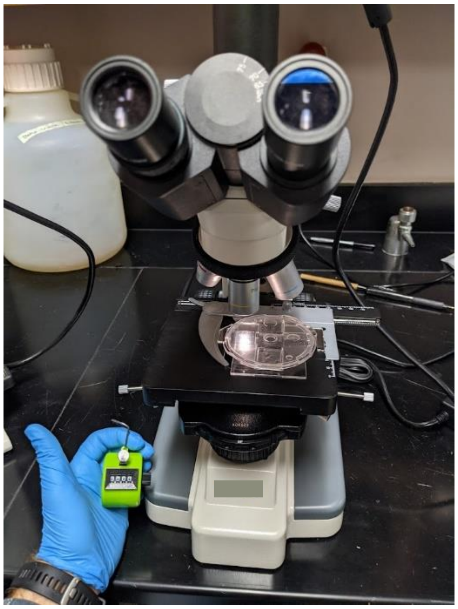

9. Enumerate soil transmitted helminth ova by carefully scanning each row in both discs. See text S4 to determine presumptive viability of observed ova.

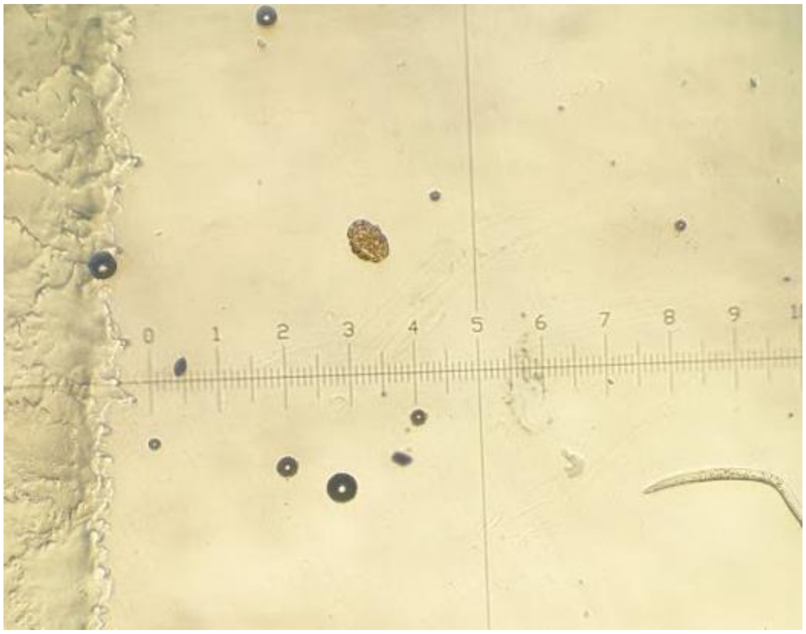
0
1
2
3
4
5
6
7
8
9

Figure S1. MapSan Trial Area

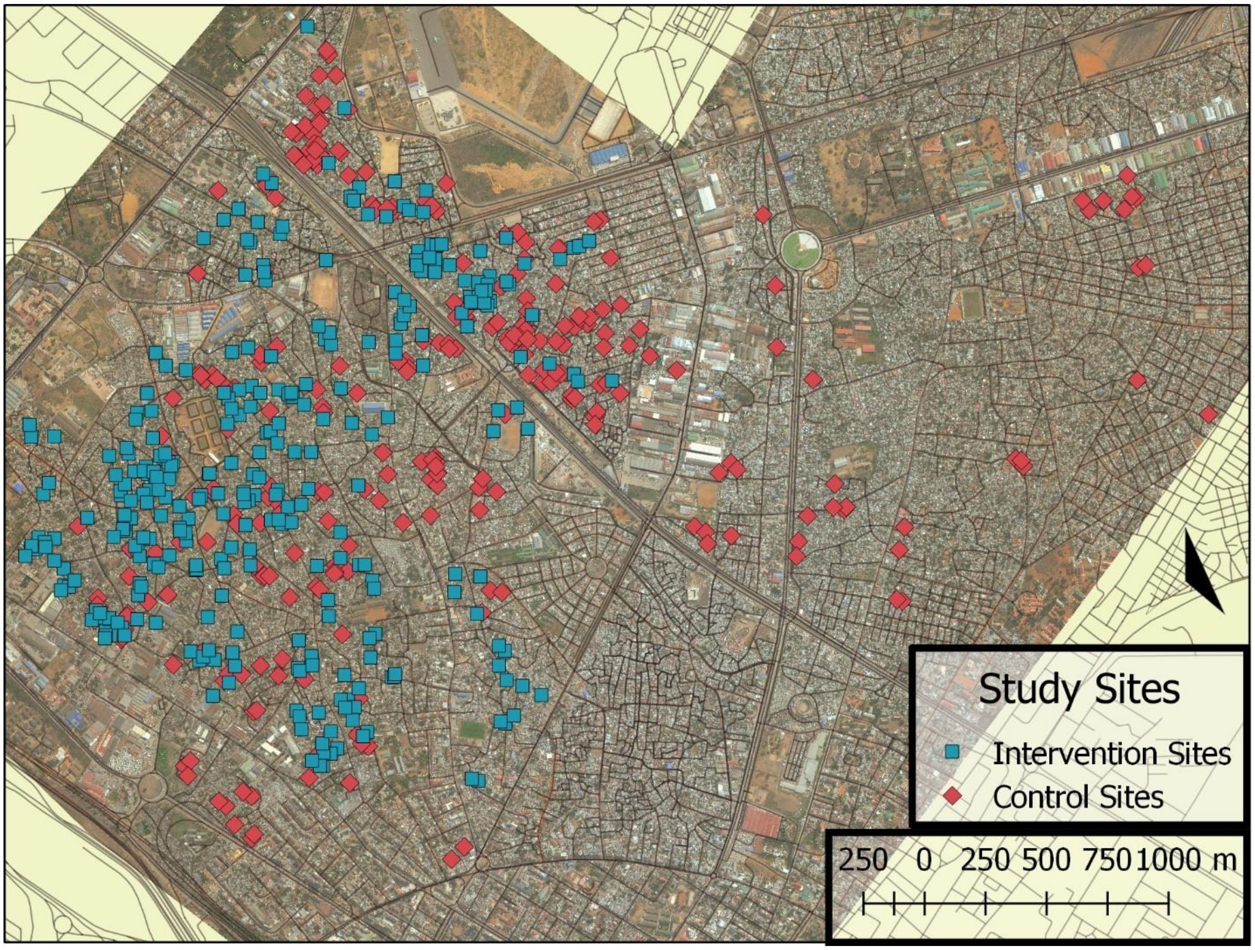

Note: Figure reproduced from Knee *et al*. 2021 (Appendix 1—figure 9, https://doi.org/10.7554/eLife.62278)

Pig farms were not present in the study area or adjacent neighborhoods. Small-holder pig operations may be found across Maputo Bay in Catembe District and north of the study area in the Zimpeto and Costa do Sol Districts. These districts are >3 kilometers from the study area. Commercial pig farms may be found outside the city in the towns of Boane, Moamba, Namaacha, and Manhiça.

Figure S2. Empirical and simulated ova in soil

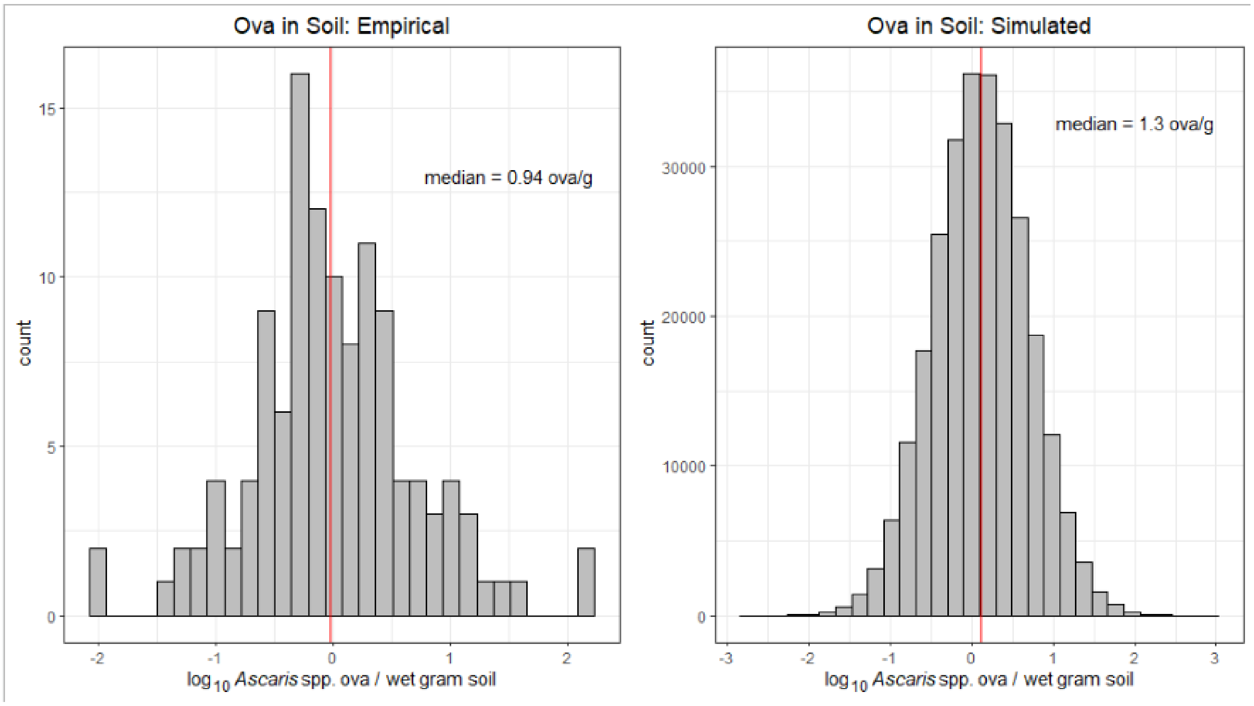

Text S6. Fecal Sludge Microscopy Protocol

1. Vortex fecal sludge cryovial for 3 seconds
2. Tare a sterile 15 mL centrifuge tube
3. Pipet or scoop 500 µL of fecal sludge from the cryotube into the sterile 15 mL tube
4. Record the mass of the fecal sludge added to the 15 mL tube (to the nearest mg)
5. Add 10 mL of $NaNO_3$ (specific gravity 1.25) to the 15 mL tube
6. Shake the tube for 20 seconds
7. Pipet the liquid from the 15 mL tube to fill 3 FLOTAC disks
8. Wait 10 minutes
9. Turn the disks using the key
10. Read the disks using microscopy at 100X and record the results

Text S7. Presumptive *Ascaris* Viability Assessment

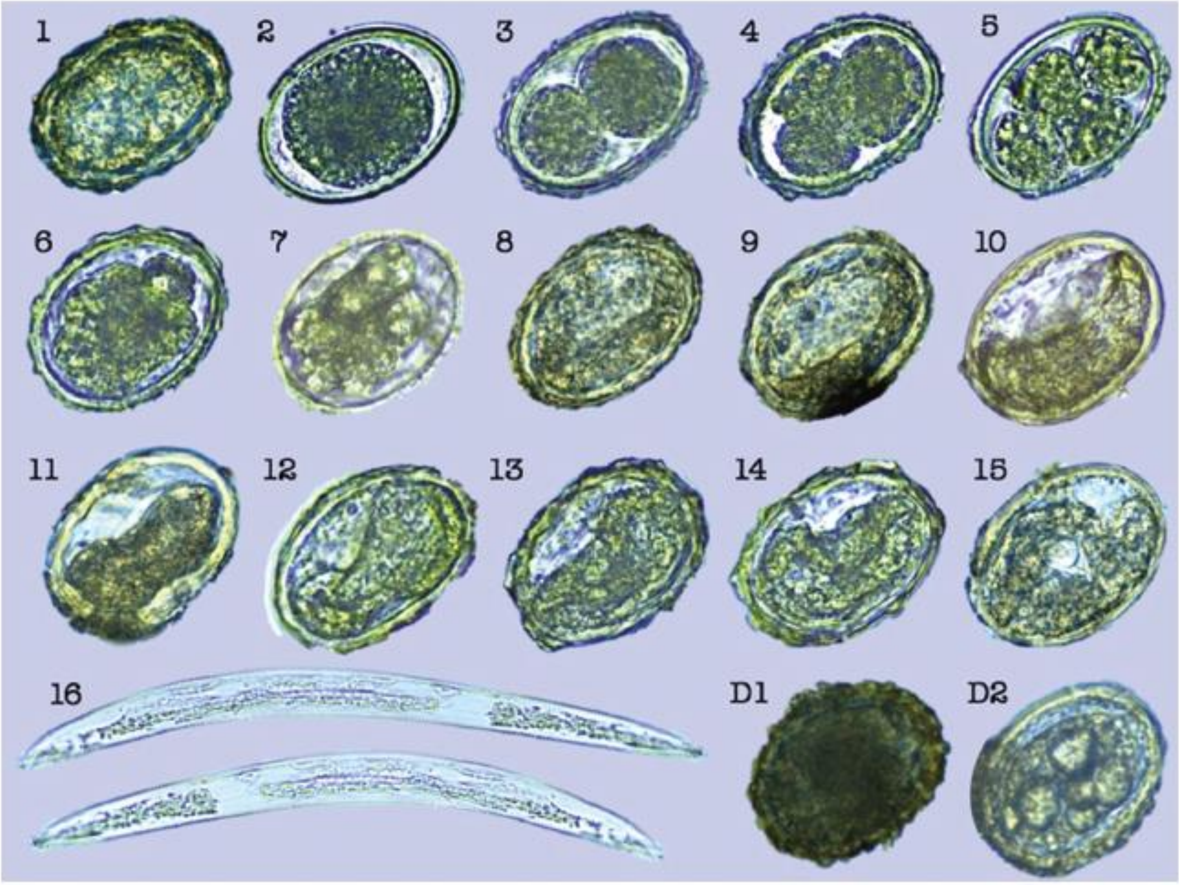

Figure 1. *A. suum ova development-stage chart for classifying ova. Unembryonated; stage 1; Embryonated, stages 2 – 7; Well-developed, stages 8 – 15; Excystation, stage 16. Dead/non-viable A. suum ova; stage D1 (disfigured dark-oval structure) and/or D2 (bubbled yolk from heat inactivation).*

We used Figure 1. from Schmitz *et al*. 2016 to estimate presumptive *Ascaris* viability based on the morphological characteristics of the ova observed.

**State**	**Identifier in Figure 1**
Viable	2-15
Non-viable	1, D2
Dead	D1
Unfertilized	Non pictured above. See below

***Ascaris lumbricoides* unfertilized eggs.**

Fertilized and unfertilized *Ascaris lumbricoides* eggs are passed in the stool of the infected host. Fertilized eggs are are rounded and have a thick shell with an external mammillated layer that is often stained brown by bile. In some cases, the outer layer is absent (known as decorticated eggs). Fertile eggs range from 45 to 75 µm in length. Unfertilized eggs are elongated and larger than fertile eggs (up to 90 µm in length). Their shell is thinner and their mammillated layer is more variable, either with large protuberances or practically none. Unfertile eggs contain mainly a mass of refractile granules.

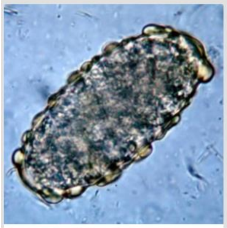

Figure A: Unfertilized egg of *A. lumbricoides*. Note the prominent mammillations on the outer layer.

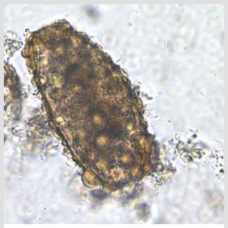

Figure B: Unfertilized egg of *A. lumbricoides* in an unstained wet mount, 200x magnification.

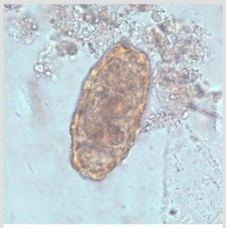

Figure C: Unfertilized egg of *A. lumbricoides* in an unstained wet mount of stool.

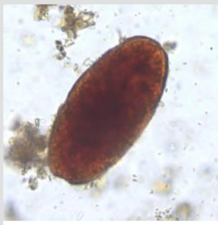

Figure D: Unfertilized egg of *A. lumbricoides* in a wet mount of stool. Note this specimen lacks the mammillated layer (decorticated).

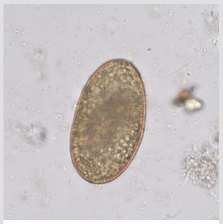

Figure E: Infertile, decorticated egg of *Ascaris lumbricoides*. Image courtesy of The Leiden University Medical Center, The Netherlands.

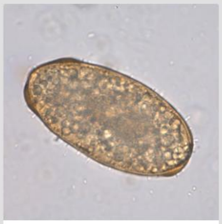

Figure F: Infertile, decorticated egg of *Ascaris lumbricoides*. Image courtesy of The Leiden University Medical Center, The Netherlands.

These are examples of unfertilized *Ascaris* ova taken from www.cdc.gov/dpdx/ascariasis/

Figure S3. Equation for Decay Constant *k*

The data used to develop the equation for the decay constant *k* were taken from Senecal *et al*. 2020.

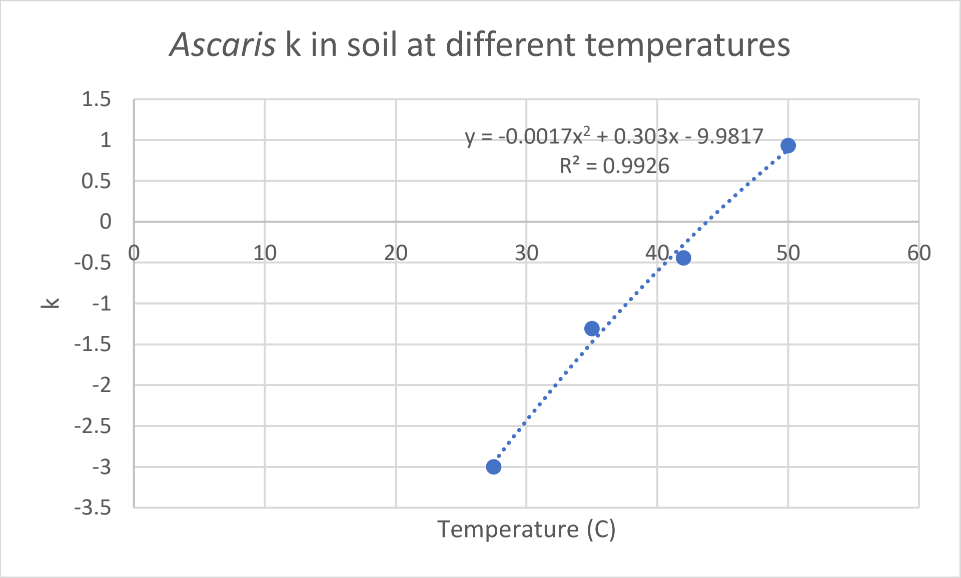

Figure S4. Empirical and simulated ova in fecal sludges

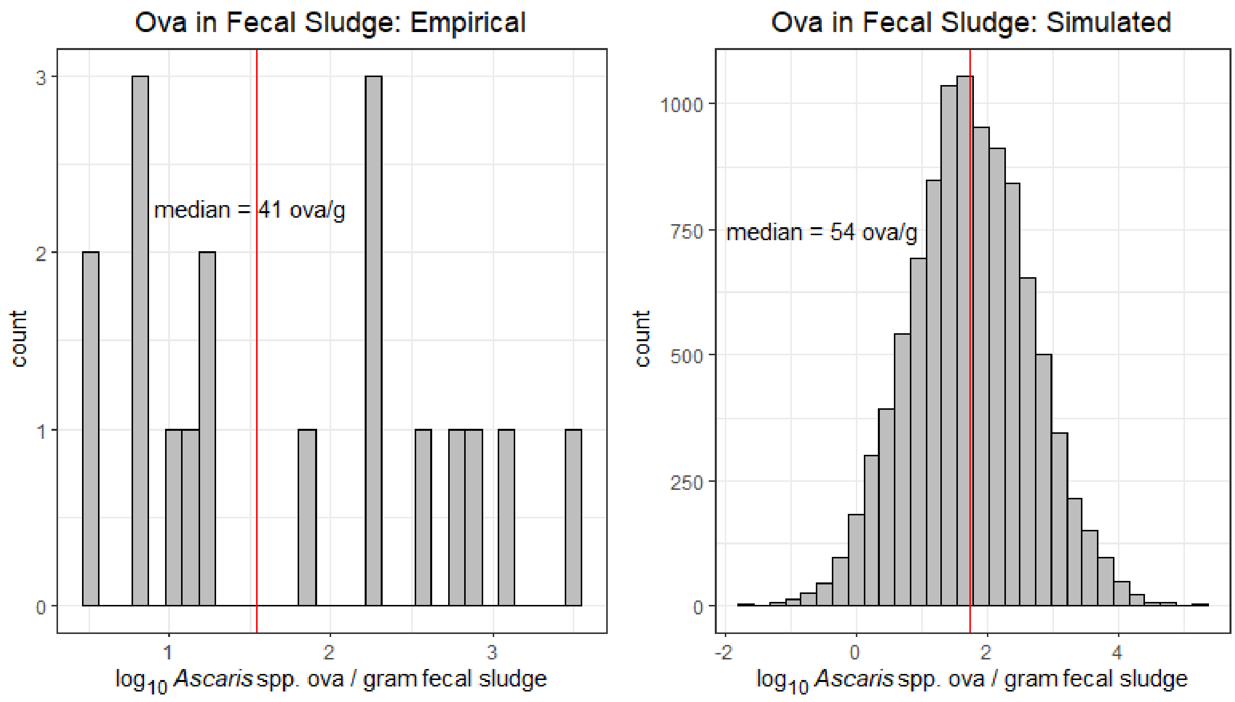

Figure S5. Empirical and simulated ova in stool

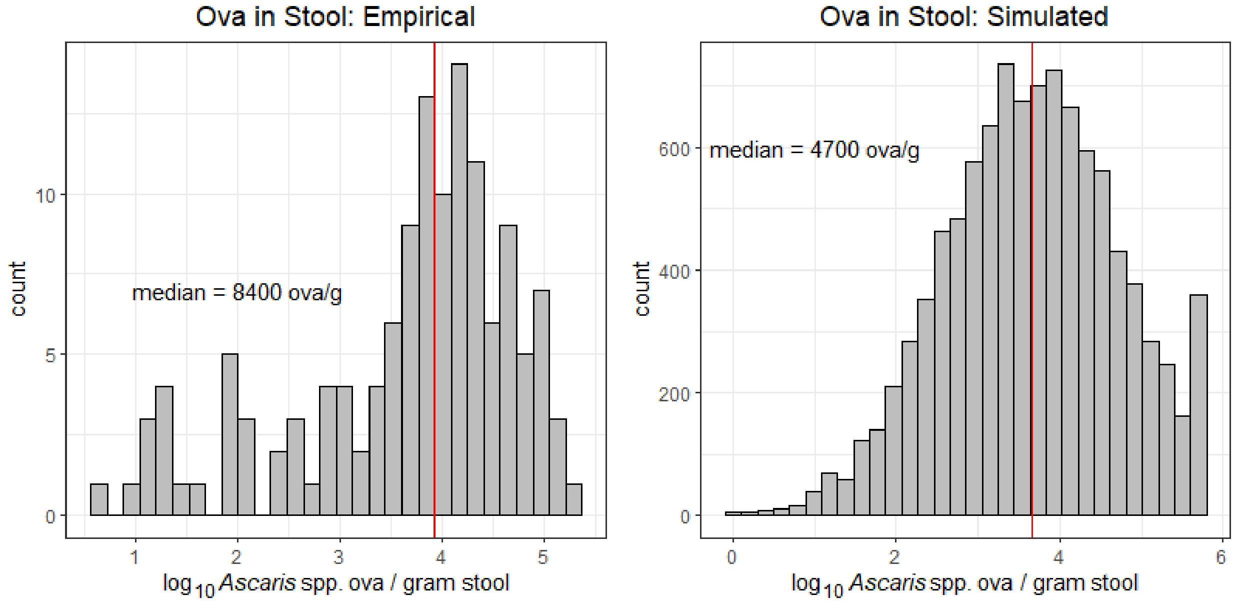

Table S1. Model Parameters

**Model variable**	**Parameters used**	**Reference**
**Variables used to define the system boundary (i.e., the hypothetical compound)**		
Median compound surface area (m^2)	124	GIS mapping, this study
Median proportion of compound covered by soil	26%	GIS mapping, this study
Maximum depth contaminated by *Ascaris* ova (cm)	0.5	Assumption based on Collender *et al*. 2015 and Storey *et al*. 1985[3,5]
Density of soil (g/cm^3)	1.7	USDA[6]
Recovery efficiency of *Ascaris* ova from sandy soil	43%	This study, Vincente *et al*. 2006[9]
Limit of detection of helminth ova in fecal sludges (ova / wet gram)	3.5	This study
Limit of detection of helminth ova in soil (ova / wet gram)	0.58	This study
**Variables used in the Monte Carlo Simulation**		
Probability of children's stool (ages 1-60 months) containing *Ascaris* ova	25%	Knee et al. 2021
Limit of detection of helminth ova in stools (ova / wet gram)	24	Kato-Katz method
Probability of fecal sludge containing *Ascaris*	88%	Capone et al. 2020
Ascaris ova density in stool from infected children (ova/ wet gram)	LN(8.4, 2.5)*<br><br>*Values were truncated at 10^0 and $10^{5.7}$ ova/gram	MLE, this study
Ascaris ova density in soil (ova/wet gram)	LN(0.26, 1.3)	MLE, this study
Ascaris ova density in fecal sludge (ova/wet gram)	LN(4.0, 2.1)	MLE, this study
Dilution factor from fecal sludge to aqueous effluent	0.055	Manga *et al*. 2017[10]
Ascaris decay rate *k* as a function of the daily average air temperature (T)	$\log_{10} k(T) = -0.0017T^2 + 0.303T - 9.9817$	Best fit binomial to data in Senecal *et al*. 2020
Average daily air temperature (T) for each day in Maputo,	Static input for each day	https://www.ncdc.noaa.gov/

Mozambique (6/1/2015 – 5/31/2018)		
Seed value in R	31	Reported for reproducibility

LN: natural log where parameters correspond to inputs of the *rlnorm* function in R

Table S2. Comparison of ova counts in soil

Variable	Reference	Unadjusted Log_{10} Difference in Ova Count (95% Confidence Interval)
Household entrance	Activity Area	-0.03 (-0.34, 0.28)
Latrine entrance		0.38 (0.05, 0.70)
Solid waste storage area		0.51 (0.18, 0.83)

Note: We conducted mixed-effects regression using the *lme4*[11] package in R. The exposure variable was compound location and the outcome variable was log_{10} transformed *Ascaris* ova concentration per wet gram soil. Non-detects were randomly imputed from 0.01 ova per gram to the LOD.

Figure S6. *Ascaris* concentration by matrix

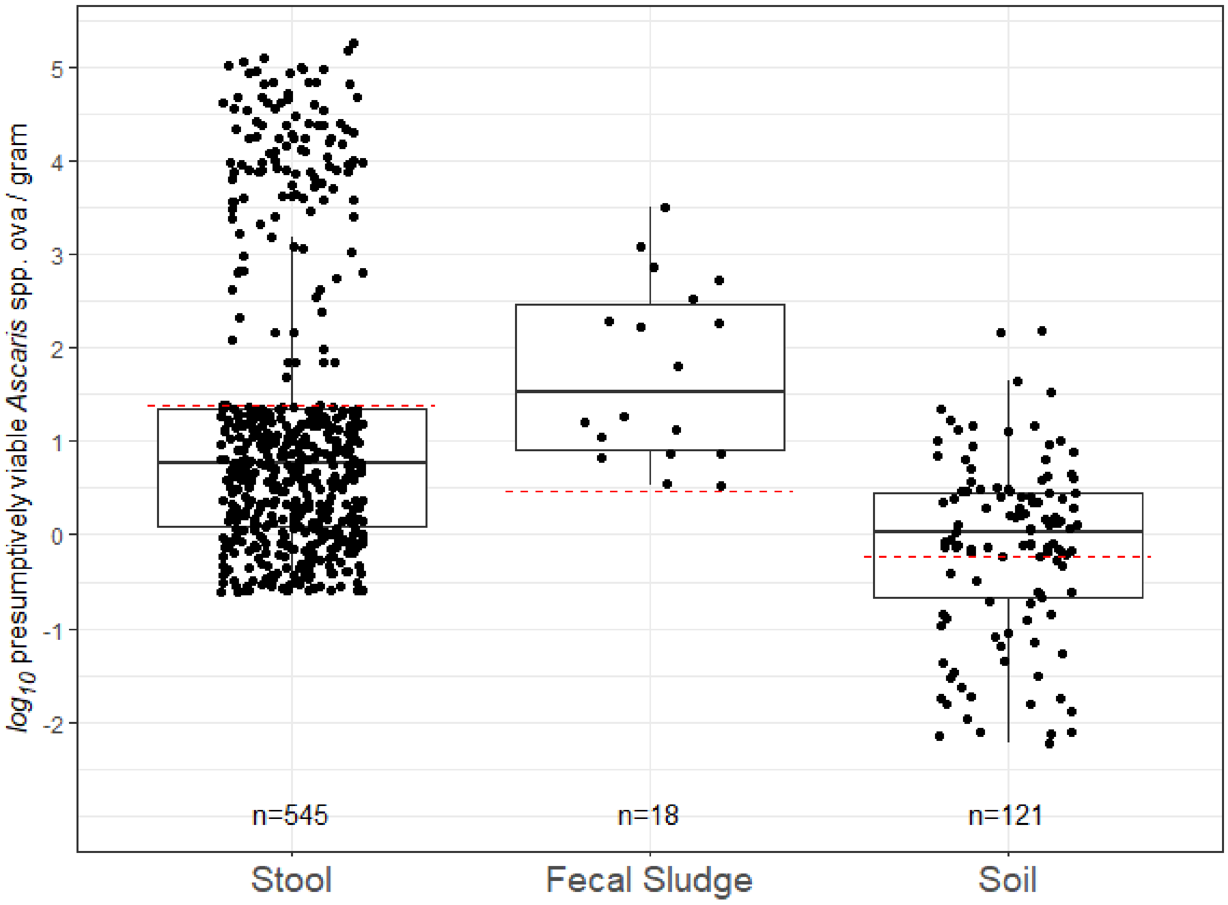

Concentration of presumptively viable *Ascaris* ova by matrix (empirical data). The dashed red lines represent the limit of detection for each matrix. Non-detects were imputed up to 2 log_{10} below the LODs to visualize sample sizes. Fecal sludge samples were randomly selected from a set of samples that had tested positive for *Ascaris lumbricoides* via PCR.

Table S3. Sensitivity Analysis

**Scenario 1 and 2**		50th percentile estimate	
Variable	Fixed value	Scenario 1 (stool, grams/day)	Scenario 2 (sludge, grams/transport event)*
Baseline values	NA	1.9	17,000
Soil depth	0.25 cm	0.90	8,600
	1.0 cm	4.0	34,000
Soil density	1.4 g/cm^3 (loosely packed)	1.5	14,000
	2.0 g/cm^3 (densely packed)	2.3	20,000
Ascaris ova recovery efficiency	16% (Silt)	5.4	46,000
	77% (Loam)	1.0	9,600
Fecal sludge transport frequency	1 year	1.9	16,000
	2 years	1.9	16,000
Viable *Ascaris* ova in soil	½ observed	0.90	8,600
	2x observed	4.0	34,000
Viable *Ascaris* ova in fecal sludges	½ observed	1.9	8,600
	2x observed	1.9	34,000
**Scenario 3**			
Variable	Fixed value	Scenario 3 (stool, grams/day)	Scenario 3 (sludge, grams/transport event)*
Percentage of daily ova die-off replaced by ova from child feces	1%	5.0×10^{-4}	17,000
	10%	6.7×10^{-3}	17,000
	50%	0.13	17,000
	90%	1.1	9,500
	99%	1.8	1,300
	100%	1.9	0
**Scenario 4**			
Variable	Fixed value	Scenario 4 (effluent, grams/day)	Scenario 4 (effluent, grams/transport event)
Dilution ratio from fecal sludge	0.01 (dilute effluent)	15,000	NA
	0.055 (baseline)	2,700	NA
	0.20 (concentrated effluent)	750	NA

*Baseline transport frequency is on day 1095

Figure S7. Ova in the system over time

This figure illustrates the total ova in the modeled compound soil if no child feces transport occurs for three years. The modeled fecal sludge emptying event would occur on the final day of the figure, bringing the quantity of ova back to the initial number of ova in the system on day 0.

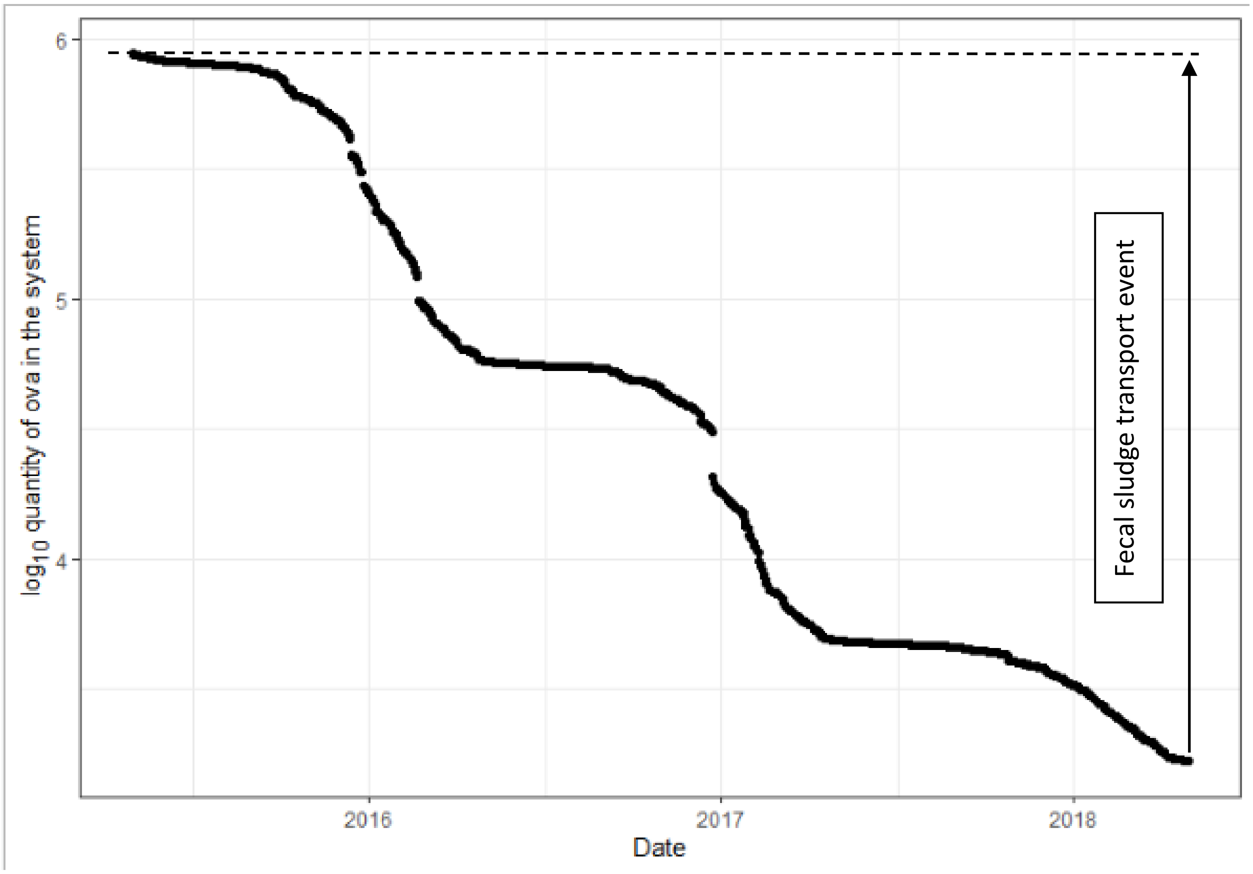

Day	Estimated ova in the system	% Reduction
0	$10^{5.94}$	0%
365	$10^{4.76}$	93.4%
730	$10^{3.69}$	99.4%
1094	$10^{3.22}$	99.8%
1095	$10^{5.94}$	0%

Figure S8. Chicken or ducks present

There was no difference in presumptively viable *Ascaris* ova concentrations in soils from compounds that had chickens or ducks present and those that did not.

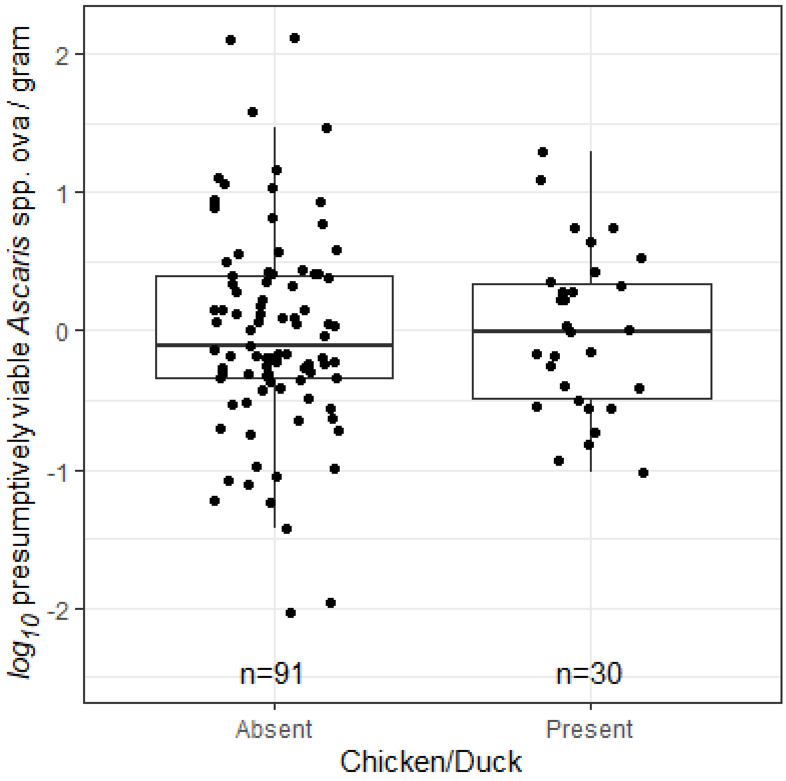

Figure S9. Dogs or cats present

There was no difference in presumptively viable *Ascaris* ova concentrations in compounds that had dogs or cats present and those that did not.

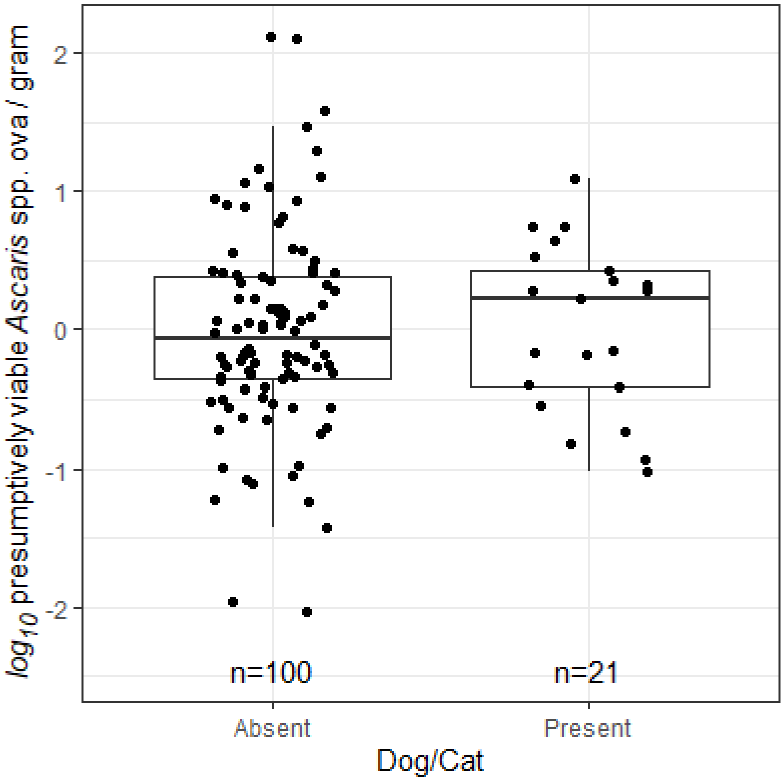

References

(1) Walker, M.; Hall, A.; Basáñez, M.-G. Individual Predisposition, Household Clustering and Risk Factors for Human Infection with Ascaris Lumbricoides: New Epidemiological Insights. *PLoS Negl. Trop. Dis.* **2011**, *5* (4), e1047. https://doi.org/10.1371/journal.pntd.0001047.

(2) Knee, J.; Sumner, T.; Adriano, Z.; Anderson, C.; Bush, F.; Capone, D.; Casmo, V.; Holcomb, D. A.; Kolsky, P.; MacDougall, A.; Molotkova, E.; Braga, J. M.; Russo, C.; Schmidt, W. P.; Stewart, J.; Zambrana, W.; Zuin, V.; Nalá, R.; Cumming, O.; Brown, J. Effects of an Urban Sanitation Intervention on Childhood Enteric Infection and Diarrhea in Maputo, Mozambique: A Controlled before-and-after Trial. *Elife* **2021**, *10*. https://doi.org/10.7554/eLife.62278.

(3) Collender, P. A.; Kirby, A. E.; Addiss, D. G.; Freeman, M. C.; Remais, J. V. Methods for Quantification of Soil-Transmitted Helminths in Environmental Media: Current Techniques and Recent Advances. *Trends Parasitol.* **2015**, *31* (12), 625–639. https://doi.org/10.1016/j.pt.2015.08.007.

(4) Vermúdez, M.; Álvarez, M.; Puertas, J.; Peña, E.; Araneda, R. J. Effects of the 2016-2018 Drought on Pequenos Libombos Reservoir (Maputo, Mozambique): A Comparative Analysis. *11th World Congr. Water Resour. Environ. Manag. Water Resour. a Sustain. Futur. - EWRA 2019.* **2019**.

(5) Storey, G. W.; Phillips, R. A. The Survival of Parasite Eggs throughout the Soil Profile. *Parasitology* **1985**, *91* (3), 585–590. https://doi.org/10.1017/S003118200006282X.

(6) USDA Natural Resources Conservation Service. Soil Quality Indicators https://www.nrcs.usda.gov (accessed Jun 25, 2021).

(7) Price, D. L. *Procedure Manual for the Diagnosis of Intestinal Parasites*; CRC Press, 2017.

(8) Ash, L. R.; Orihel, T. C. *Human Parasitic Diseases: A Diagnostic Atlas*; ASCP Press, 2020.

(9) Vicente, E. M.; Jermy, C. A.; Schreiner, H. D. Urban Geology of Maputo, Mocambique. In *10th Congress of the International Association for Engineering Geology and the Environment*; London Geological Society: London, 2006.

(10) Manga, M. The Feasibility of Co-Composting as an Upscale Treatment Method for Fecal Sludge in Urban Africa, University of Leeds, United Kingdom, 2017.

(11) Bates, D.; Machler, M.; Bolker, B.; Walker, S. Fitting Linear Mixed-Effects Models Using {lme4}. *J. Stat. Softw.* **2015**, *67*, 1–48. https://doi.org/10.18637/jss.v067.i01.
